# Low-dose phase retrieval of biological specimens using cryo-electron ptychography

**DOI:** 10.1038/s41467-020-16391-6

**Published:** 2020-06-02

**Authors:** Liqi Zhou, Jingdong Song, Judy S. Kim, Xudong Pei, Chen Huang, Mark Boyce, Luiza Mendonça, Daniel Clare, Alistair Siebert, Christopher S. Allen, Emanuela Liberti, David Stuart, Xiaoqing Pan, Peter D. Nellist, Peijun Zhang, Angus I. Kirkland, Peng Wang

**Affiliations:** 1 National Laboratory of Solid State Microstructures, Jiangsu Key Laboratory of Artificial Functional Materials, College of Engineering and Applied Sciences and Collaborative Innovation Center of Advanced Microstructures, Nanjing University, Nanjing, 210093 China; 2 State Key Laboratory of Infectious Disease Prevention and Control, National Institute for Viral Disease Control and Prevention, Chinese Center for Disease Control and Prevention, Beijing, 100052 China; 3 Department of Materials, University of Oxford, Parks Road, Oxford, OX1 3PH UK; 4 Electron Physical Sciences Imaging Centre, Diamond Light Source Ltd., Harwell Science and Innovation Campus, Didcot, OX11 0DE UK; 5 The Rosalind Franklin Institute, Harwell Campus, Didcot, OX11 0FA UK; 6 Division of Structural Biology, Wellcome Trust Centre for Human Genetics, University of Oxford, Oxford, OX3 7BN UK; 7 Electron Bio-Imaging Centre, Diamond Light Source, Harwell Science and Innovation Campus, Didcot, OX11 0DE UK; 8 Department of Materials Science and Engineering, and Department of Physics and Astronomy, University of California, Irvine, CA 92697 USA

**Keywords:** Cryoelectron microscopy, Cryoelectron microscopy, Transmission electron microscopy

## Abstract

Cryo-electron microscopy is an essential tool for high-resolution structural studies of biological systems. This method relies on the use of phase contrast imaging at high defocus to improve information transfer at low spatial frequencies at the expense of higher spatial frequencies. Here we demonstrate that electron ptychography can recover the phase of the specimen with continuous information transfer across a wide range of the spatial frequency spectrum, with improved transfer at lower spatial frequencies, and as such is more efficient for phase recovery than conventional phase contrast imaging. We further show that the method can be used to study frozen-hydrated specimens of rotavirus double-layered particles and HIV-1 virus-like particles under low-dose conditions (5.7 e/Å^2^) and heterogeneous objects in an Adenovirus-infected cell over large fields of view (1.14 × 1.14 μm), thus making it suitable for studies of many biologically important structures.

## Abstract (web summary)

Cryo-electron microscopy is widely employed in structural biology and uses phase contrast imaging. Here, the authors employ electron ptychography, a quantitative phase retrieval method for high-contrast, low-dose phase imaging of cryo-state rotavirus and immature HIV-1 virus-like particles, and show that electron ptychography is more efficient for phase recovery than conventional phase contrast imaging.

## Introduction

The need to determine the three-dimensional (3D) structures of biological macromolecules and assemblies at high resolution in their native states has motivated significant and sustained efforts to develop electron microscopy (EM) techniques, most notably phase contrast cryo-EM^1–6^. However, unstained biological samples embedded in thin vitreous ice are essentially pure phase objects that are extremely radiation sensitive^2^, and consequently images of these have low signal-to-noise ratios^7^ and low contrast^8,9^. To counter the latter, high defocus values can be used but these corrupt information transfer in single images at intermediate and high spatial frequencies due to rapid oscillations in the phase contrast transfer function^10^. Despite these limitations, by suitably averaging large numbers of homogeneous objects and using direct electron detectors^11^ to improve the signal-to-noise ratio, single particle analysis can reconstruct 3D biological structures at close to atomic resolution^12,13^. Averaging however, is more challenging for heterogeneous samples, specimens at low concentration, low symmetry structures and small (<250 kDa), or flexible molecules^14,15^. For these reasons, methods that improve information transfer over a wide range of spatial frequencies are required. One solution is to introduce a phase shifting device in the back focal plane of the objective lens and both Zernike^8^ and Volta phase plates^9^ which introduce a phase shift between the transmitted and scattered electrons have been fabricated and are commonly used for this purpose. However, these devices can suffer from signal attenuation at high frequencies, inconsistent fabrication, poor reliability and short working lifetimes due to electrostatic charging^16,17^.

Ptychography is an alternative method based on scanning diffraction microscopy, as originally proposed by Hoppe^18^. This approach uses a probe to illuminate the sample and records a series of far-field diffraction patterns as a function of probe position to recover the sample exit plane wavefunction using one of several iterative^19,20^ or direct methods^19,21,22^. Ptychography has been most widely used in light and X-ray optics^23–25^ as the wavefunction recovered can exceed the spatial resolution that can be obtained using conventional optics. In EM, it has also attracted considerable interest for its potential application in super-resolution imaging^21,26^, high-contrast light-element detection^27–29^, 3D optical sectioning^29,30^ and coupling to spectroscopic data acquisition^31^. Recently reported simulations have compared the performance of ptychography using both iterative Bayesian recovery with a defocused probe^32^ and single side band recovery with a focused probe^33^ to conventional phase contrast TEM showing that ptychography gives a higher signal to noise ratio for a finite dose and improved resilience to the effects of partial coherence. For inorganic materials atomic-resolution low-dose ptychography has been demonstrated for a two dimensional MoS_2_ crystal using direct electron detectors that can record low noise data at low electron doses^34^. Hence, due to its efficient phase recovery, robustness under low electron dose conditions^34^ and the quantitative recovery of the 3D wavefunction^30,35^, electron ptychography has significant potential for application in the structure determination of biological samples^32,33,36^. An important additional advantage of this method is the ability to tune information transfer to maximize low or high spatial frequencies by altering the probe convergence angle.

In this work, we report the results of cryo-electron iterative ptychography (cryo-EPty) of biological structures. Our ptychographic phase reconstructions of rotavirus double-layered particles (DLPs) and non-symmetric immature HIV-1 virus-like particles (VLPs) in a cryo-state show excellent phase sensitivity and improved information transfer particularly at a low spatial frequencies under low dose conditions. We further examine ptychographic data acquisition using data recorded at room temperature collected from resin-embedded Adenovirus-infected cells, which demonstrates that this method can also be applied to micron wide fields of view.

## Results

### Cryogenic ptychographic reconstruction

**Fig. 1:**
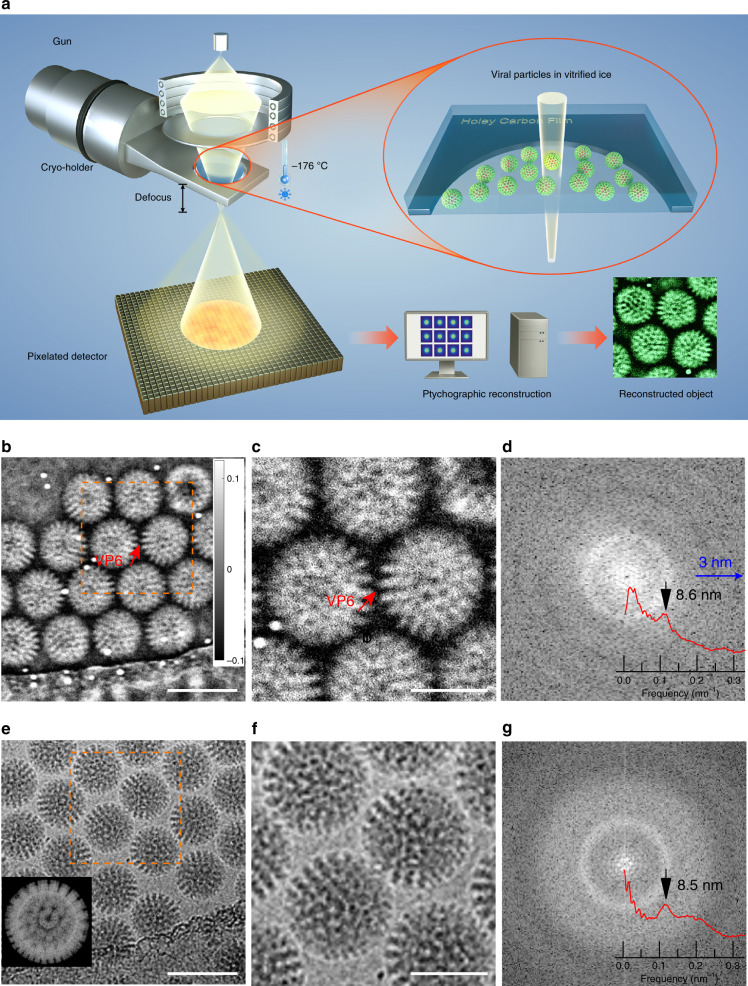
Cryo-ptychographic configuration and reconstruction of rotavirus DLPs. **a** Schematic diagram of the optical configuration. **b** Ptychographic reconstructed phase at a dose of 22.8 e/Å^2^ and **e** defocused TEM images at dose of 35 e/Å^2^ of rotavirus DLPs in vitrified ice. **c**, **f** Magnified views of sub areas marked in **b** and **e** with orange squares, respectively. The red arrows in **b** and **c** indicate the capsid trimers of viral protein 6 (VP6). The ~6 nm gold particles are displayed as bright features in **b** and **c** following established convention where a phase advance (+) is displayed as white^53^ and appears as a contrast reversal compared to conventional TEM images. **d**, **g** Power spectra calculated from **b** and **e**, respectively including radial averages (inset). A rotavirus DLP model is shown in **e** (inset). The gray scale bar in **b** is in radians and applies to **b** and **c**. Scale bars: 100 nm in **b** and **e**, 50 nm in **c** and **f**.

Electron ptychographic data was recorded in a scanning electron diffraction mode as shown schematically in Fig. 1a at 300 kV using a double aberration corrected JEOL ARM300CF. An electron probe with a convergence semi-angle of 1.03 mrad, a defocus of −13.0 µm and a diameter of 26.9 nm was used. Ptychographic datasets were acquired on a 256 × 256 pixel Merlin Medipix3^37^ direct electron detector with the probe rastered across the sample. Details of the experimental settings used are given in Supplementary Table 1 (Setting 1) and additional details of the experimental protocols are provided in Materials and Methods and Supplementary Note 1. Rotavirus DLPs with a diameter of 76.5 nm and immature HIV-1 VLPs with variable sizes were used to demonstrate this method under cryo-conditions^38^. Both samples were vitrified in liquid ethane using standard procedures, transferred into the microscope and kept at liquid N_2_ temperature using a Gatan 698 Elsa cryo-holder. Further details of the cryo-sample preparation are given in Supplementary Note 2.

The phase and amplitude recovered using the ptychographic iterative algorithm (ePIE)^20^ (See details in Methods) of the rotavirus DLPs at a dose of 22.8 e/Å^2^ are shown in Fig. 1b, c and Supplementary Fig. 1, respectively. At this dose, the phase shows strong contrast from the virus particles, where both the capsid trimers of viral protein 6 (VP6) and the channels between these can be clearly seen as shown in the enlarged image in Fig. 1c. The observation of these features and the resolution of the viral capsid’s symmetric elements are consistent with those observed using conventional defocused TEM images collected at a dose of 35 e/Å^2^ distributed over 29 fractions (Fig. 1e, f) and with the known 3D structure (PDB ID 3KZ4) (inset to Fig. 1e). In order to compare information transfer using ptychography and conventional phase contrast imaging at high defocus, power spectra and corresponding radial averages were calculated (Fig. 1d, g). In general, the power spectrum calculated from a ptychographic reconstruction is continuous over its entire spatial frequency range and does not show Thon rings, arising from zero-crossings in the phase contrast transfer function (CTF), as observed in single phase contrast TEM images at high defocus. The former also shows strong information transfer at an intermediate spatial frequency of 0.116 nm^−1^ corresponding to the VP6 trimers separated by a distance of about 8.6 nm and at lower spatial frequencies extending down to 76.5 nm, which correspond to the contrast defining the overall shape of the virus particles. This demonstrates that ptychography can recover phase information from biological structures with high contrast over a larger bandwidth of spatial frequencies without contrast reversals.

### Contrast transfer in electron ptychography

Unlike phase contrast TEM, the ptychographic phase is reconstructed in silico and the ePIE algorithm used therefore controls the resulting information transfer. These algorithms also contain implicit noise filtering which also varies with spatial frequency. To illustrate this we use simulated ptychographic phases to calculate the transfer for a weak phase object as a function of spatial frequency for the electron-optical conditions and algorithm used. This demonstrates the key information transfer features of ptychographic reconstruction. However, a detailed general discussion of the ptychographic signal-to-noise ratio as a function of spatial frequency^39^ is beyond the scope of this work.

**Fig. 2:**
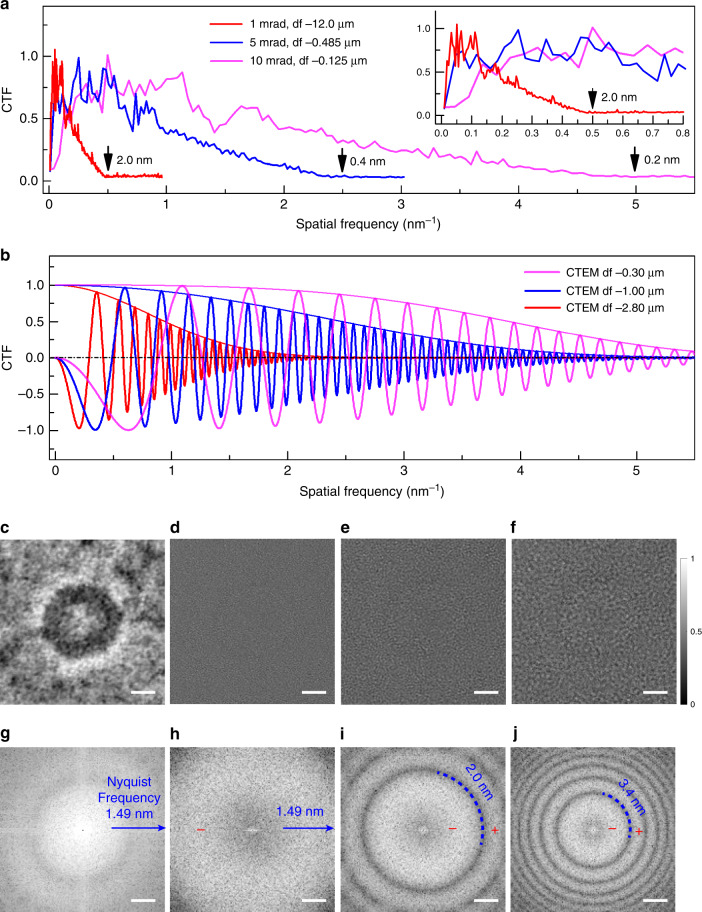
CTFs and comparisons of ptychography and defocused TEM. **a** Simulated ptychographic phase CTFs for different convergence semi-angles with Poisson noise; 1 mrad (red line), 5 mrad (blue line), 10 mrad (magenta line) at an accelerating voltage of 80 kV. **b** Conventional phase CTFs of noise free for images simulated at different defoci: −0.3 µm (magenta line), −1.0 µm (blue line), −2.8 µm (red line) using envelope functions calculated according to refs. ^54–56^ at an accelerating voltage of 80 kV. **c** Experimental reconstructed ptychographic phase at dose of 146 e/Å^2^ and TEM images at defoci of **d** −0.32 μm, **e** −1.0 μm, **f** −2.8 μm at dose of 180 e/Å^2^, of a resin embedded Adenovirus particle recorded at room temperature. For comparison, the ptychographic phase and TEM images are displayed normalized to a range of 0 to 1. Power spectra calculated from **g** the ptychographic phase and **h** to **j** TEM images. Additional calculation parameters are given in Supplementary Tables 2 and 3. The reconstructed ptychographic phase and CTEM images are both displayed normalized to a range of 0 to 1 corresponding to the greyscale on the right of **f**. Scale bars: 25 nm in **c** to **f** and 0.2 nm^−1^ in **g** to **j**.

To demonstrate how information transfer is affected by the convergence semi-angle, numerically calculated phase CTFs are shown in Fig. 2a based on reconstructions of a ptychographic dataset using a model of a two dimensional amorphous thin film with convergence semi-angles of 1, 5 and 10 mrad at 80 kV and including Poisson noise. For comparison, phase CTFs for conventional defocused TEM images without noise at 80 kV were also calculated with defoci of −0.30, −1.0 and −2.8 µm, respectively as shown in Fig. 2b. Further details of these calculations and the model used are given in Supplementary Note 3 and Supplementary Fig. 2 and 12 with corresponding simulation parameters given in Supplementary Tables 2 and 3.

In comparison to phase contrast TEM imaging, the phase CTFs for ptychographic reconstruction (Fig. 2a) have no zero-crossings, consistent with the experimental observations shown in Fig. 1d, g and with experimental results discussed later. Overall, the ptychographic reconstruction is not affected by contrast reversals over the entire spatial frequency range recovered and consequently, the resulting data can be interpreted directly and intuitively.

In ptychography, the transfer function scales with respect to spatial frequency as a function of the convergence angle falling to zero at a spatial frequency corresponding to 2*α*/*λ*^40^, where *α* is the convergence semi-angle and *λ* is the wavelength. This therefore defines the resolution limit for the technique. As the convergence semi-angle decreases, the bandwidth of the CTFs is shifted from high to low spatial frequencies (Fig. 2a) for a given accelerating voltage. This is a significant advantage for ptychography in that the CTF can be tuned to enhance information transfer within a particular spatial frequency range. For example, in virus particles with diameters between 50 and 100 nm, the spatial frequencies defining the overall shape lie between 0.02–0.01 nm^−1^ and hence the CTF for a convergence semi-angle of 1 mrad provides more efficienct transfer in this range than those for 5 and 10 mrad. These calculations show that using a lower convergence semi-angle provides better contrast at the low spatial frequencies needed to observe the overall virus morphology. As in phase contrast TEM, defocus also enhances low spatial frequency transfer^36^, which is a further benefit of the defocused ptychography mode used here. However, this results in some weak oscillations in the CTF as can be seen in Fig. 2g and Supplementary Fig. 2, but unlike phase contrast TEM, defocus does not generate zero crossings nor contrast reversals.

### Tunable bandwidth information transfer

To further demonstrate the tunable bandwidth of the ptychographic CTF, a systematic series of room temperature datasets using a sample of resin-embedded Adenovirus particles at convergence semi-angles of 1.37, 4.68 and 10 mrad were recorded at 80 kV. Further details of the relevant experimental settings and sample preparation are provided in Supplementary Table 4 (Settings 1–3) and Supplementary Note 2. The spatial frequency corresponding to the outline of the Adenovirus particles (80–100 nm in diameter) lies between 0.0125–0.01 nm^−1^, and therefore the phase CTFs shown in Fig. 2a (and corresponding power spectrum in Fig. 2g) reconstructed using a 1.37 mrad convergence semi-angle at 80 kV should show the enveloping viral capsid shape with maximum phase contrast as shown in Fig. 2c.

Information transfer at low spatial frequencies was explored using the phase of the reconstructed complex wavefunction of an Adenovirus particle at convergence semi-angles of 1.37, 4.68 and 10 mrad (Supplementary Fig. 3a–c). The reconstructed phase at 1.37 mrad shows the overall shape of the particle with high contrast; the core-shell structure with a hexagonal shell matching the capsid coating and a triangular core corresponding to the internal genome are both partially resolved. This observation is consistent with the model of the Adenovirus (PDB ID 6GCV) shown in Supplementary Fig. 3d. Furthermore, a line profile extracted across the reconstructed phase shows variations within the core-shell structure (Supplementary Fig. 3e), confirming strong transfer of phase information at low spatial frequencies.

Radially averaged power spectra (Supplementary Fig. 3g) show that for convergence semi-angles larger than 1.37 mrad, information transfer at spatial frequencies below 0.029 nm^−1^ (34 nm) is reduced, resulting in low contrast for features corresponding to the overall shape of the particles (Supplementary Fig. 3b, c). To verify this the reconstructed phase shown in Supplementary Fig. 3a was band pass-filtered using three frequency windows at 0.0–0.029, 0.029–0.410 and 0.410–0.949 nm^−1^ as shown in Supplementary Fig. 4b–d, which demonstrates that the overall shape of the particles is only preserved in the low spatial frequency band (Supplementary Fig. 4b). Therefore, for a given object size (80–100 nm for Adenovirus), the information transfer can be tuned by changing the convergence semi-angle to maximize the reconstructed phase over a particular spatial frequency range.

To evaluate resolution (the high spatial frequency band) of the ptychographic reconstruction, Fourier ring correlation (FRC)^41,42^ was used, which measures the degree of correlation between two images as a function of spatial frequency. To calculate the FRC, the full data shown in Supplementary Fig. 3a–c was split into two independent datasets (Supplementary Fig. 3f), which were used for independent reconstruction. Details of the FRC analysis are provided in Supplementary Note 4 and the resulting FRCs are shown in Supplementary Fig. 3h with resolutions estimated using a ½-bit threshold criterion^42^ of 1.49, 0.62 and 0.51 nm for convergence semi-angles of 1.37, 4.68 and 10 mrad.

The thin biological samples used in this work are weakly scattering objects^43^ giving rise to low signal-to-noise ratio at high angles under low dose conditions. The theoretical resolution limits for convergence semi-angles of 1.37, 4.68 and 10 mrad are 1.5, 0.45 and 0.20 nm which are slightly higher than those calculated from FRCs. However, splitting of the dataset reduces the probe overlap ratio and the overall dose in the two sub-datasets, which will decrease the quality of the ptychographic reconstruction. Hence, the FRC gives a conservative estimate of the spatial resolution.

### Conventional phase contrast TEM

For comparison with the ptychographic reconstructions, conventional phase contrast TEM (CTEM) images containing multiple resin-embedded Adenovirus particles were acquired from the same sample using a similar dose at across a wide range of defoci from −0.32 μm to −25 μm as shown in Supplementary Fig. 5. CTEM imaging parameters are provided in Supplementary Note 5. CTEM images of individual particles recorded at defoci of −0.32, −1.0 and −2.8 μm are shown in Fig. 2d, e and f, respectively, with their corresponding power spectra shown in Fig. 2h, i and j. The inherent compromise in this imaging mode between overall contrast and the transfer of high spatial frequencies is clearly visible in the CTEM images and their corresponding CTFs. The CTEM image at small defocus (Fig. 2d) preserves high spatial frequencies up to 1.49 nm, but the overall contrast of the virus is so low that it is not visible. At a defocus of −2.8 μm, the contrast improves (Fig. 2f), although the virus is barely visible without sequential CTF correction procedures^44^ and the first zero crossing of the CTF is limited to 3.4 nm (Fig. 2j). Therefore, as expected although phase contrast can be improved by using large defoci, this limits information transfer at intermediate and high spatial frequencies^10^. In contrast, ptychographic reconstruction offers advantages in that it allows more efficient phase retrieval and retains strong information transfer over a wider spatial frequency bandwidth.

### Low dose cryo-electron ptychography

**Fig. 3:**
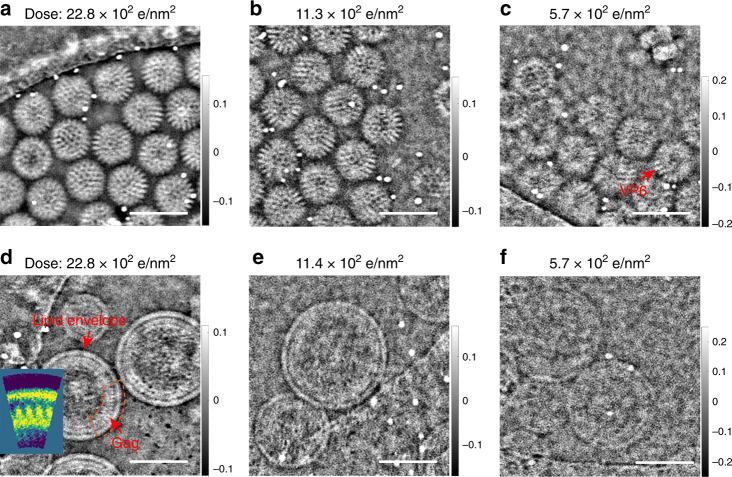
Low-dose cryo-ptychographic reconstruction of viral particles. Reconstructed phase of rotavirus DLPs at doses of **a** 22.8 e/Å^2^, **b** 11.3 e/Å^2^ and **c** 5.7 e/Å^2^. The red arrow in **c** indicates the VP6 trimers. Reconstructed phase of non-symmetrical immature HIV-1 VLPs at doses of **d** 22.8 e/Å^2^, **e** 11.4 e/Å^2^ and **f** 5.7 e/Å^2^. The red arrows in **d** indicate the HIV-1 Gag protein and the lipid envelope. Inset to **d** is a rotational average of the sub-region indicated by the orange dashed line, (for details see Supplementary Fig. 10 and Supplementary Note 7). Scale bars: 100 nm.

To be generally applicable to structural studies of biological materials in a cryo state, ptychographic reconstruction must be effective under low dose conditions similar to those used for CTEM imaging of these materials. Defocused probe ptychography uses a relatively large probe (25–30 nm in diameter for the experiments reported here) and hence it is possible to scan a large sample area with a small number of probe positions, reducing the total dose if the overlap between neighboring probe positions is optimized^34^. Figure 3a–c show ptychographic phase reconstructions of rotavirus DLPs embedded in vitrified ice for doses between 22.8 e/Å^2^ and 5.7 e/Å^2^, with the reconstructed amplitude and calculated power spectra shown in Supplementary Fig. 6. Further details of the relevant experimental settings are provided in Supplementary Table 1 (Settings 1–3). Even at a dose of 5.7 e/Å^2^ (Fig. 3c), the viral capsid shape and contrast from the VP6 trimers remain visible (also see the line profiles in Supplementary Fig. 6m). Similarly, Fig. 3d–f shows the ptychographic phase of immature HIV-1 VLPs in vitrified ice for different doses. At a dose of 22.8 e/Å^2^ (Fig. 3d), the lipid envelope, the N-terminal domain and C-terminal domain of the capsid part of the Gag protein are clearly resolved in the immature HIV-1 VLPs corresponding to the structural model^45^, and at a dose of 5.7 e/Å^2^, the contrast from different subunits in the VLPs is still recognizable (Fig. 3f, Supplementary Fig. 7).

### Phase retrieval over large areas

**Fig. 4:**
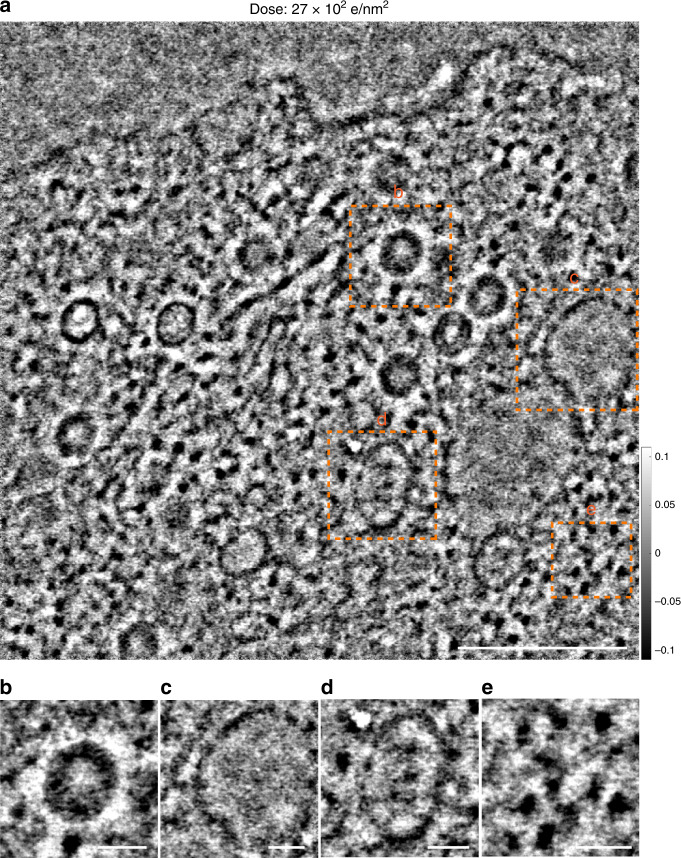
Low-dose large scale ptychographic reconstruction at room temperature. **a** Micrometer scale reconstructed phase of an Adenovirus-infected cell recorded at a dose of 27 e/Å^2^. Magnified views of **b** a viral particle, **c** a vacant vesicle. **d** A transport vesicle and **e** free ribosomes taken from regions indicated with orange squares in **a**. Scale bars are **a** 300 nm, and **b**–**e** 50 nm.

The final requirement for the application of ptychographic reconstruction in structural and cellular biology is the ability to recover information from large fields of view corresponding to cellular ultrastructures. To demonstrate this a micrometer (1.14 × 1.14 μm) area of a resin-embedded Adenovirus-infected cell was reconstructed at a dose of 27 e/Å^2^ (Fig. 4a and Supplementary Fig. 8a) using Setting (4) in Supplementary Table 4. The ptychographic phase shows good visibility of ultrastructural features of varied size, including cytoskeletal elements, viral particles (Fig. 4b), vacant vesicles (Fig. 4c), transport vesicles (Fig. 4d) and free ribosomes (Fig. 4e). Moreover, as already noted, reconstruction of the ptychographic phase preserves low spatial frequency information, facilitating the location of these key molecular features in a cellular context.

The redundancy and overlap ratio of the reconstruction shown in Fig. 4a is 191 and 77%, (250 × 250 scanning positions) (Supplementary Table 4). This suggests that the dose can be futher reduced by decreasing the overlap ratio and hence the total number of scanning positions used in the reconstruction. As an example, if the overlap ratio is decreased to 66.5% or 44.2%, the number of scanning positions are decreased to 170 × 170 and 102 × 102, respectively with corresponding redundancies of 86 and 31. The resultant dose required for reconstruction is then futher reduced from 27 e/Å^2^ to 12 e/Å^2^ and 4 e/Å^2^, respectively as shown in Supplementary Fig. 8b, c. However, as expected the contrast reduces as the dose decreases. This shows that in the low dose regime, Poisson noise plays a dominant role as there are insufficient electrons scattered into each pixel above the noise level to provide measureable interference in the diffraction patterns. Therefore, ultimately the image contrast is dose limited, consistent with the work of Song et al.^34^.

## Discussion

Despite the current highly advanced state of cryo-EM using conventional phase contrast CTEM imaging, the major limitation to resolution is radiation sensitivity and low contrast due to low-atomic number elements. Here, we have described an alternative method (ptychography) that can provide more efficient recovery of phase information, with better sensitivity and improved information transfer from larger structural features under low dose conditions in a cryo state. These advantages of ptychography over phase contrast CTEM are attributed to a number of factors. Firstly, its quantitative, high phase sensitivity, provides accurate structural information for materials cointaining light atoms^27,29^. Secondly, for a given dose, ptychography shows higher phase contrast than CTEM in agreement with recent theoretical predictions, where the signal-to-noise ratio of defocused iterative ptychography using Bayesian optimization^32^ at a dose of 20 e/Å^2^ is predicted to be two orders of magnitude better than that of phase contrast CTEM. The greater resilience of ptychography to the effects of partial coherence also provides a further improvement in the signal-to-noise ratio^33^. Practically, ptychography allows virtual post-acquisition focusing^30^ which provides data at an optimal defocus. This can potentially contribute to an additional saving in the overall dose budget by eliminating refocusing of the sample during either sample searching or image collection. Thirdly, compared to phase contrast CTEM, ptychography has a wider, tunable and continuous CTF using either iterative approaches (Fig. 2a) or direct solutions^40^ for weak phase objects. The lack of zero crossings in the CTF eliminates contrast reversals with varying defocus providing directly interpretable phase data. The band pass spatial frequencies of the CTF can also be shifted by adjusting the convergence angle such that low and high spatial frequency information can be recovered in a controlled way. By reducing the convergence angle as experimentally demonstrated here, the reconstructed phase (Fig. 1b) is enhanced at low spatial frequencies, which is important for imaging many biological structures. Fourthly, as ptychography uses a scanning geometry it is capable of potentially recording unlimited fields of view without a loss in spatial resolution^46^. Finally, ptychography using an inverse multi-slice method^30^ can computationally section z-slices of interest from thicker biological samples, where the projection approximation breaks down as a consequence of significant multiple scattering within the sample. Furthermore, unlike CTEM images taken with a µm diameter stationary beam ptychography uses a spot scan imaging geometry. This acquisition geometry reduces the area of the specimen that is illuminated at any given time and may alleviate beam induced motion of the specimen due to charge accumulation^47,48^, which has been reported as a factor affecting image quality in conventional cryo-EM^49^. Therefore, given the advantages above, ptychography can be considered as a competitive alternative to defocused phase contrast cryo-EM imaging as conventionally used in structural biology and in some areas might be expected to outperform the latter.

In future, we anticipate further developments, most notably, combining ptychography with single particle analysis for 3D reconstruction of small macromolecules, with particular applications to low symmetry and low molecular weight structures or to larger scale molecular architectures in individual cells and multicellular organisms. The tunable information transfer will also enable resolution extension beyond that achievable using conventional TEM images at high defoci. One possible approach to using this potential would be to firstly acquire high resolution data with a large convergence semi-angle as higher spatial information is lost earlier^50^ during electron beam exposure and then to capture low spatial frequency data with a smaller convergence semi-angle to map low resolution features in a partly damaged structural landscape.

In conclusion, we have reported cryo-electron iterative ptychography demonstrating high contrast quantitative phase recovery at an electron dose of 5.7 e/Å^2^ over wide fields of view making this approach suitable for biological macromolecular imaging. This new approach provides tunable, continuous wide-band information transfer including low spatial frequencies that are inaccessible using conventional phase contrast imaging. Efficient phase recovery using ptychography provides higher signal-to-noise data than phase contrast imaging in cryo-TEM, which potentially reduces the particle numbers required for 3D reconstruction, facillitating 3D classification of heterogeneous specimens at low concentration or from structures with low symmetry. Using an inverse multi-slice method^35^ ptychography can recover 3D optically sectioned phase data from thick samples^30,51^ which could be used to reject sections containing ice but no sample. Finally, for use in electron tomography, the ptychographic phase should improve the data quality captured at each tilt angle and hence expand the applicability of cryo-electron tomography to thicker samples and larger sample volumes^52^.

## Methods

### Samples

Three samples were used; rotavirus DLPs, immature HIV-1 VLPs which were plunged frozen in vitrified ice and an Adenovirus-infected cell embedded in resin. Full details of the sample preparation are provided in Supplementary Note 2.

### Experimental measurements

Vitrified samples were transferred into a Gatan 698 Elsa cryo-transfer holder for cryo observation and the specimen temperature was maintained at −176 ± 2 °C during the ptychographic dataset acquisitions. A conventional single tilt holder was used for room temperature dataset acquisition from samples of an Adenovirus infected cell embedded in resin. Ptychographic datasets were recorded using a 256 × 256 pixel Medipix3^37^ direct electron detector with Merlin Readout in a scanning electron diffraction mode using a JEOL ARM 300CF operated at 300 kV (for rotavirus DLPs and immature HIV-1 VLPs in vitrified ice) and 80 kV (for Adenovirus infected cells at room temperature) using the settings given in Supplementary Tables 1 and 4, respectively. Using Setting (4) in Supplementary Table 4 as an example, the acquisition time for each DP was set to 1.3 ms and the reciprocal pixel-size of the detector was 0.023 mrad in the diffraction plane. Hence, the maximum collection semi-angle was 2.89 mrad, which is sufficiently large to record the entire bright field disk on the detector used (Supplementary Fig. 9). The schematic of the illumination optics used is given in Supplementary Fig. 11. Details of additional settings are given in Supplementary Note 1.

### Iterative ptychographic reconstruction

The ePIE algorithm^20^ was used for ptychographic reconstruction. We define a probe function, *P*, an object function,*O*, an object exit wave function*,ψ*, a diffraction pattern,ψ, the measured intensity of this diffraction pattern, *I*, coordinates in reciprocal space, *ν*, and real space, *s* and the probe position, *s*_*i*_. Using this notation a summary of the ePIE algorithm is given below.

At a probe position, *s*_*i*_, an estimate of the object exit wave function is formed as:

1 $${\it{\uppsi }}_{\it{i}}\left( {\it{s}} \right) = {\it{P}}_{\it{i}}\left( {{\it{s}} - {\it{s}}_{\it{i}}} \right){\it{O}}_{\it{i}}\left( {\it{s}} \right)$$

An initial guess of the diffraction pattern, Ψ_*i*_(*ν*) in the far field is calculated after a Fourier transform of the object exit wave function as:

2 $${\mathrm{\Psi }}_{\it{i}}\left( v \right) = {\cal{F}}\left[ {{\it{\uppsi }}_i\left( {\it{s}} \right)} \right]$$

The modulus of the above initial guess, Ψ_*i*_(*ν*) is subsitituted for the square root of the measured intensity, *I*_*i*_(*ν*) preserving the phase. Following an inverse Fourier transform, the revised diffraction pattern is back propagated to generate an updated exit wave, ${\it{\uppsi }}\prime _i\left( {\it{s}} \right)$ in real space as:

3 $${\it{\uppsi }}\prime _i\left( s \right) = {\cal{F}}^{ - 1}\left[ {\frac{{{\mathrm{\Psi }}_{\it{i}}\left( {\it{v}} \right)}}{{\left| {{\mathrm{\Psi }}_{\it{i}}} \right.\left. {\left( {\it{v}} \right)} \right|}}\sqrt {I_{\it{i}}\left( v \right)} } \right]$$

Subsequently, an updated object function, *O*_*i*+1_(*s*) and updated probe function, _*Pi*+1_(*s*) are computed as:

4 $${\it{O}}_{{\it{i}} + 1}\left( s \right) = {\it{O}}_{\it{i}}\left( s \right) + {\it{\upalpha }}\frac{{{\it{P}}_{\it{i}}^ \ast ({\it{s}} - {\it{s}}_{\it{i}})}}{{\left| {{\it{P}}_{\it{i}}(s - s_i)} \right|_{{\it{max}}}^2}}\left[ {{\it{\uppsi }}\prime _i\left( {\it{s}} \right) - {\it{\uppsi }}_{\it{i}}\left( {\it{s}} \right)} \right]$$

5 $${\it{P}}_{{\it{i}} + 1}\left( {\it{s}} \right) = {\it{P}}_i\left( {\it{s}} \right) + {\it{\upbeta }}\frac{{{\it{O}}_{\it{i}}^ \ast ({\it{s}} + {\it{s}}_{\it{i}})}}{{\left| {{\it{O}}_{\it{i}}({\it{s}} + {\it{s}}_{\it{i}})} \right|_{{\it{max}}}^2}}[{\it{\uppsi }}\prime _i\left( {\it{s}} \right) - {\it{\uppsi }}_{\it{i}}\left( {\it{s}} \right)]$$

The step size of the update is adjusted by the *α* and *β* parameters. * refers to the complex conjugate and *max* defines the maximum value of the function. The above procedure is continued until the diffraction pattern at each probe position has been used to update the probe and object functions, which then defines a single ePIE iteration. In our work, 300 iterations were run for all reconstructions. The estimated probe function used for reconstruction and reconstructed probe function are shown in Supplementary Fig. 13. More details of the reconstructions used in this work are given in Supplementary Note 6.

### Cryo conventional TEM of DLPs

Movies were acquired on a Titan Krios transmission electron microscope using a Falcon III detector operated in linear mode using a total exposure of 35 e/Å^2^ distributed over 29 fractions.

### Reporting summary

Further information on research design is available in the Nature Research Reporting Summary linked to this article.

## Supplementary information

Supplementary Information

Reporting Summary

## Data Availability

All relevant data are available from the corresponding authors upon reasonable request.

## Code availability

The ePIE algorithm code for data analysis is available from the corresponding author (P.W.) upon request.
