# Supplementary material for: Low-dose phase retrieval of biological specimens using cryo-electron ptychography
Source: Nat Commun. 2020 Jun 2;11:2773. doi: 10.1038/s41467-020-16391-6 (PMC7265480; doi:10.1038/s41467-020-16391-6)
Supplement: Supplementary file 1 — Supplementary Information [file 41467_2020_16391_MOESM1_ESM.pdf]

Supplementary Information

Low-Dose Phase Retrieval of Biological Specimens using Cryo-Electron Ptychography

Liqi Zhou, Jingdong Song, Judy S. Kim et al.

Supplementary Figures

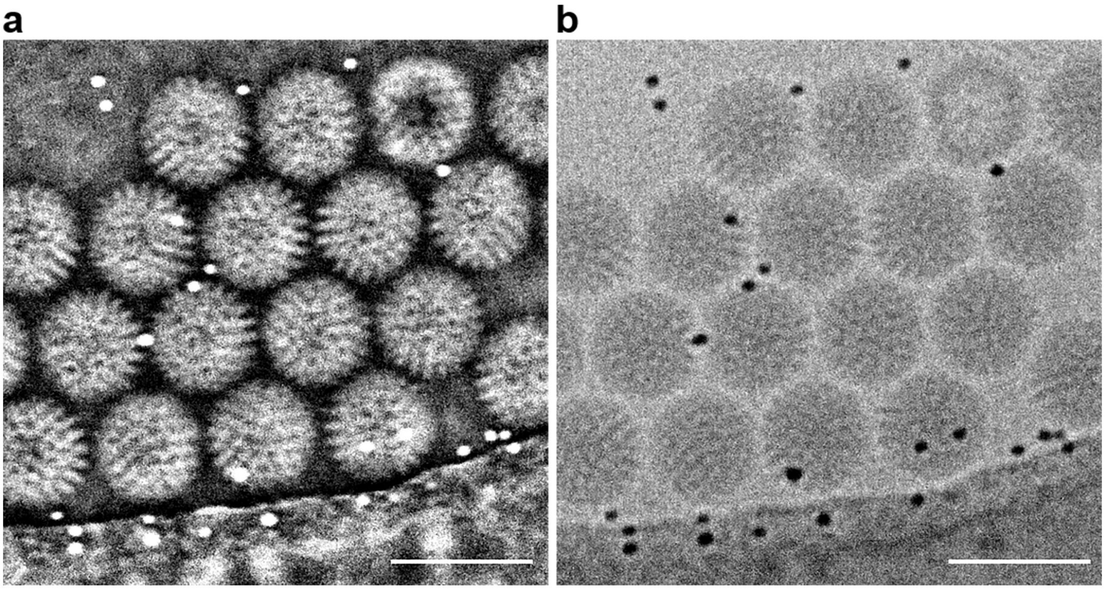

Supplementary Figure 1

Ptychographic reconstruction of rotavirus DLPs in vitrified ice at a dose of 22.8 e/Å^2.

(**a**) Phase and (**b**) amplitude of rotavirus DLPs in vitrified ice. Scale bars: 100 nm.

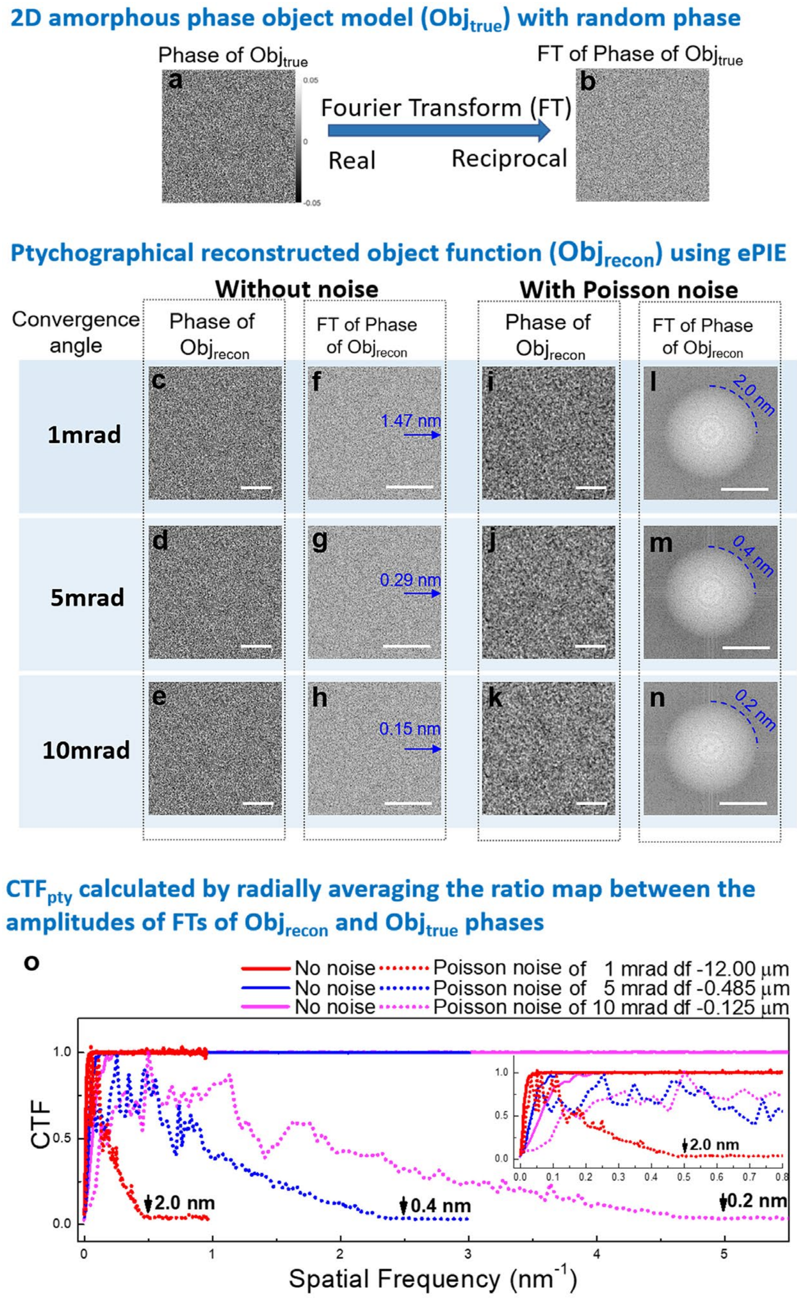

Supplementary Figure 2

Calculated CTF$_{Pty}$ for different convergence semi-angles with and without Poisson noise. (**a**) Phase of the original object function (obj$_{true}$) of a two dimensional amorphous phase model and (**b**) its corresponding power spectra. This original object function (obj$_{true}$) was used to calculate the ptychographic dataset of diffraction patterns shown in **Supplementary Fig. 12**, from which ptychographic reconstructed object functions (obj$_{recon}$) are subsequently recovered using the ePIE algorithm. (**c** to **e**) Phase of reconstructed ptychographic object functions (obj$_{recon}$) without noise for convergence

semi-angles of 1, 5, 10 mrad, respectively and (**f** to **h**) their corresponding power spectra. (**i** to **k**) Phase of reconstructed ptychographic object functions (obj_{recon}) with added Poisson noise for convergence semi-angles of 1, 5, 10 mrad, respectively and (**l** to **n**) their corresponding power spectra. (**o**) Plots of CTF_{Pty} for convergence semi-angles of 1, 5 and 10 mrad with and without Poisson noise, respectively. Plots (for instance, the curve for Poisson noise of 1mrad) were calculated by radially averaging the ratio between the amplitudes of the Fourier Transforms of the phases of obj_{recon} (**l**) and obj_{true} (**b**). Scale bars in (**c** and **i**), (**d** and **j**) and (**e** and **k**) are 50, 10 and 5 nm, and 2 mrad, 10 mrad and 20 mrad in (**f** and **l**), (**g** and **m**) and in (**h** and **n**), respectively.

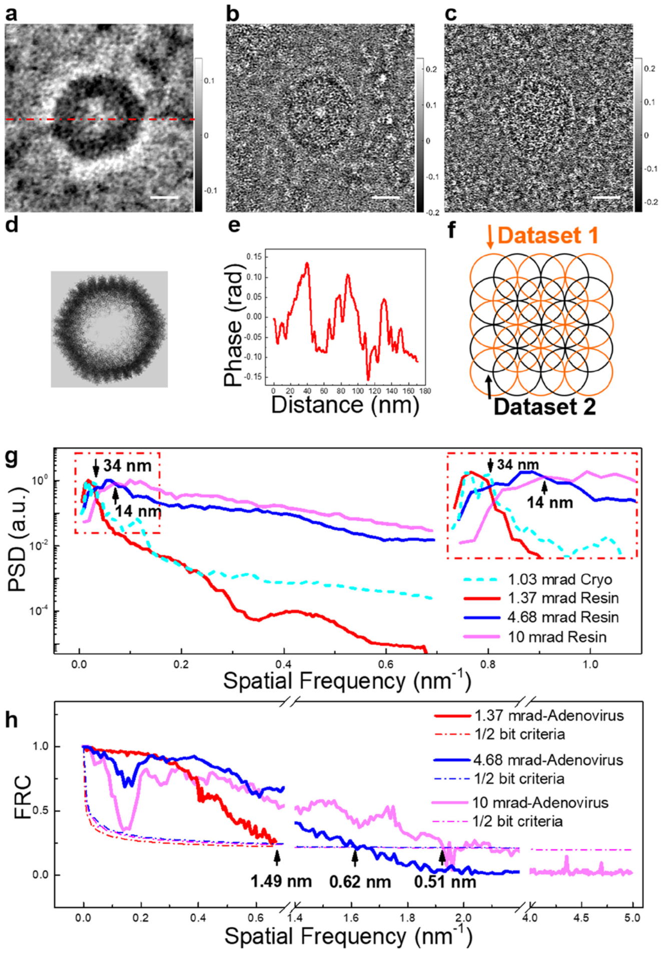

Supplementary Figure 3

Low and high spatial frequency ptychographic information transfer limits for different convergence semi-angles. (**a**, **b**, **c**) Experimental ptychographic phase of an Adenovirus with convergence semi-angles of 1.37 mrad at dose of 146 e/Å^2, 4.68 mrad at dose of 234 e/Å^2 and 10 mrad at dose of 175 e/Å^2, respectively. The size variation of the Adenovirus particle in (**a** to **c**) is due to the sample being sliced at different height. (**d**) Structure model of an Adenovirus particle. (**e**) Line profile extracted from (**a**) at the position indicated. (**f**) Schematic diagram showing a single ptychographic dataset split by selecting alternate measurements to estimate the resolution using FRC. (**g**) Radially averaged power spectra calculated from (**a to c**) and (**Fig. 1b**). **Inset** to (**g**) shows a magnified view of the area marked by the red square. (**h**) FRCs of the complex functions of (**a** to **c**). Scale bars: 25nm (**a** to **c**).

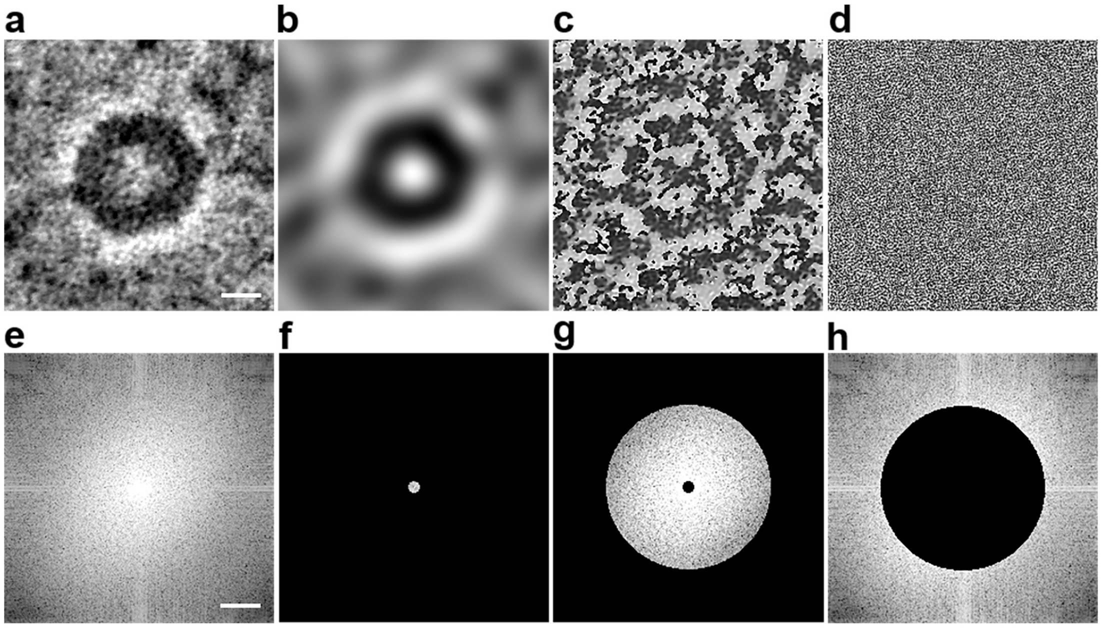

Supplementary Figure 4

Band-pass-filtered reconstructed phases of an Adenovirus for different spatial frequency ranges. (**a**) Original reconstructed phase at dose of 146 e/Å^2. (**b** to **d**) Band-pass-filtered phases calculated from (**a**) for ranges of (**b**) 0.0-0.029 nm^{-1}; (**c**) 0.029-0.410 nm^{-1}; (**d**) 0.410-0.949 nm^{-1}. (**e** to **h**) Corresponding power spectra of (**a** to **d**). Scale bars: 25 nm in (**a**), and 0.2 nm^{-1} in (**e**).

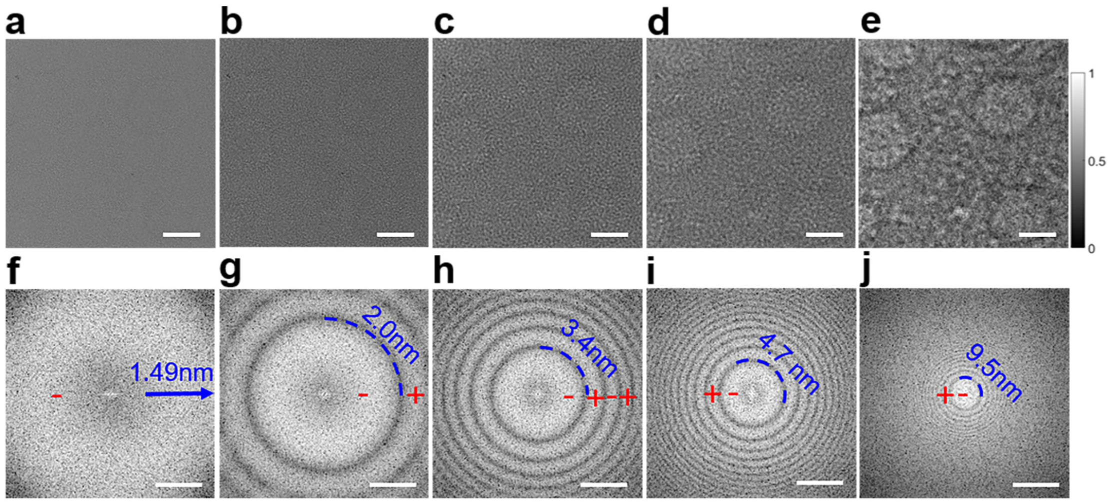

Supplementary Figure 5

Conventional defocused Phase contrast CTEM images of multiple Adenovirus particles at defoci of (**a**) -0.32 µm, (**b**) -1.0 µm, (**c**) -2.8 µm, (**d**) -5.5 µm, (**e**) -25 µm at dose of 180 e/Å^2. CTEM images are displayed normalized to a range of 0 to 1. Power spectra (**f** to **j**) calculated from TEM images. Scale bars: 25 nm in (**a** to **e**), and 0.3 nm^{-1} in (**f** to **j**).

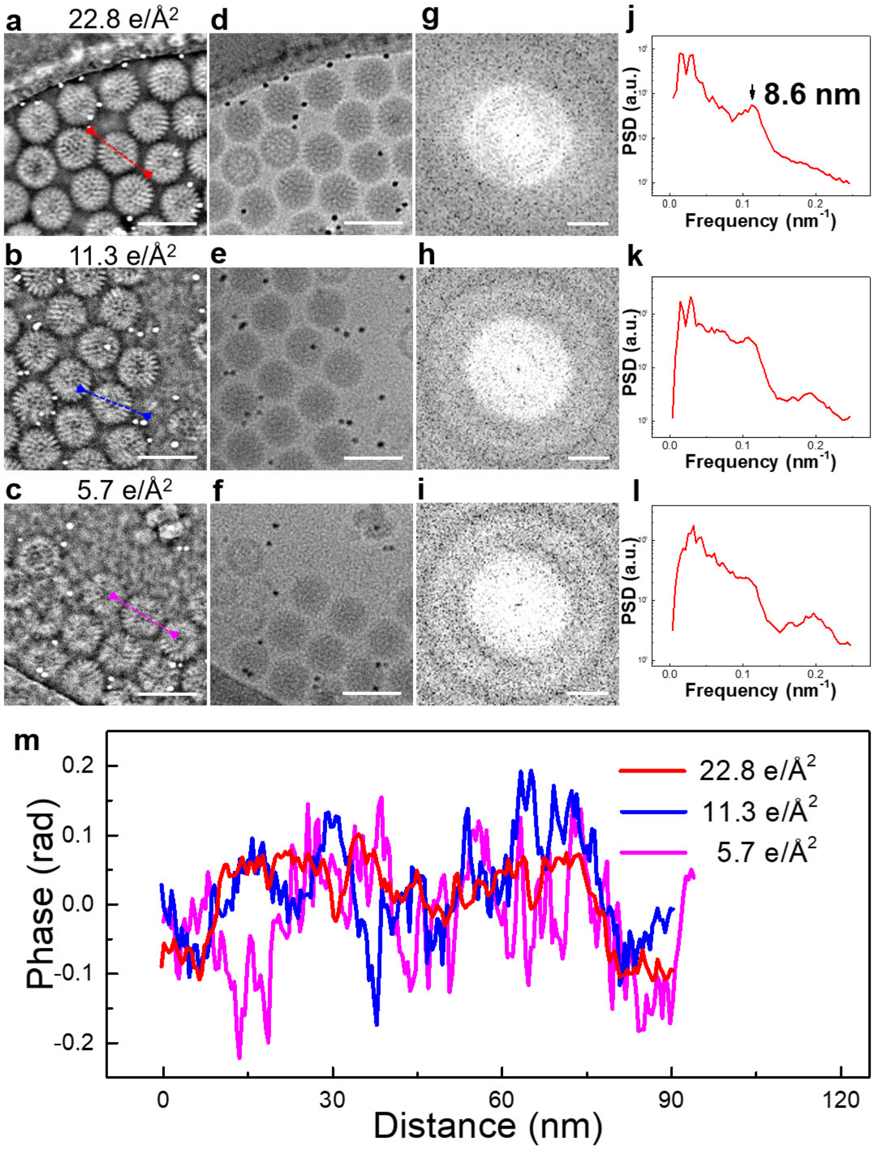

Supplementary Figure 6

Ptychographic reconstruction of rotavirus DLPs in vitrified ice at different doses. (**a** to **c**) Phase, (**d** to **f**) amplitude, (**g** to **i**) power spectra calculated from the phase and (**j** to **l**) corresponding radially averaged power spectra displayed on a log scale at doses of 22.8, 11.3 and 5.7 e/Å^2, respectively. (**m**) Line profiles of the reconstructed ptychographic phase at different doses extracted across the viral particles shown in (**a** to **c**). Scale bars: 100 nm in (**a** to **f**), and 0.1 nm^{-1} in (**g** to **i**).

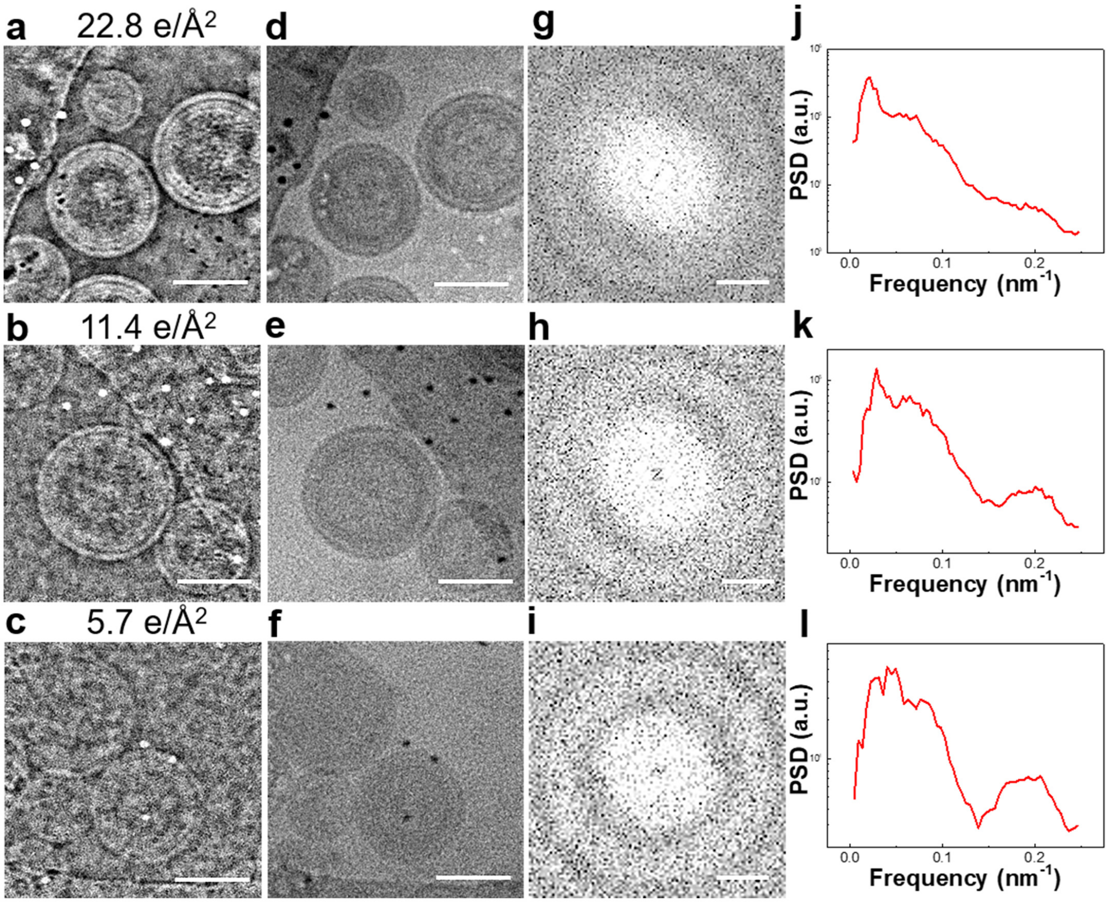

Supplementary Figure 7

Ptychographic reconstruction of immature HIV-1 VLPs in vitrified ice at different doses. (**a** to **c**) Phase, (**d** to **f**) amplitude, (**g** to **i**) power spectra calculated from the phase and (**j** to **l**) corresponding rotationally averaged power spectra displayed on a log scale at doses of 22.8, 11.4 and 5.7 e/Å^2, respectively. A structure model of immature HIV-1 VLPs can be found in Ref[16]. Scale bars: 100 nm in (**a** to **f**), and 0.1 nm^{-1} in (**g** to **i**).

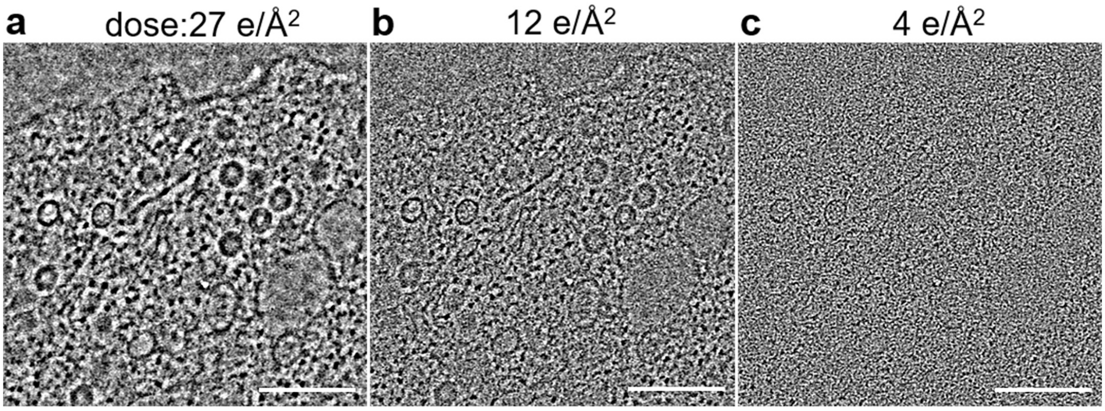

Supplementary Figure 8

Room temperature large scale ptychographic reconstruction of an Adenovirus-infected cell. Reconstructed phase at doses of **(a)** 27 e/Å^2, **(b)** 12 e/Å^2 and **(c)** 4 e/Å^2, respectively. Scale bars: **(a to c)** 300 nm.

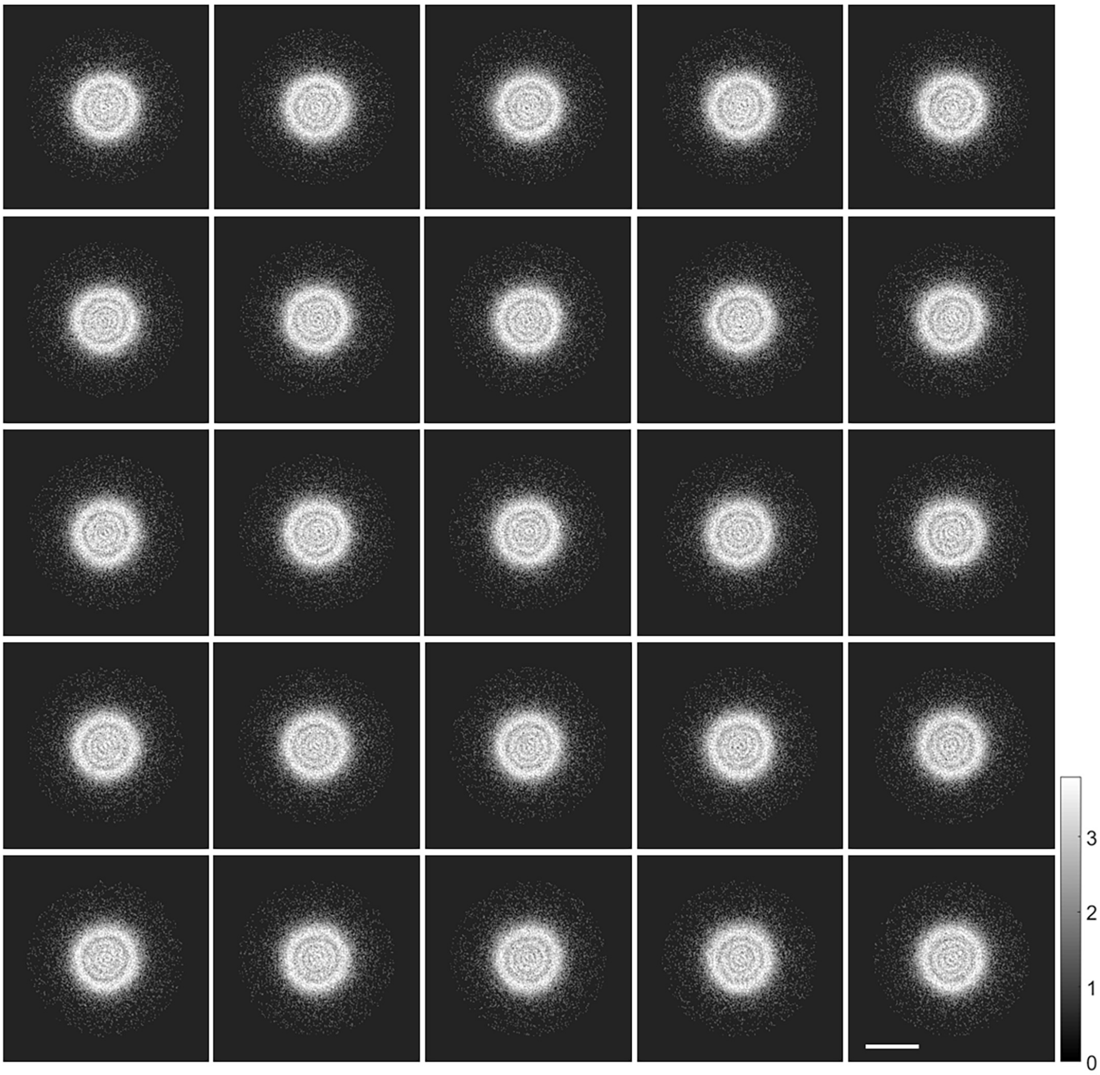

Supplementary Figure 9

Diffraction patterns in a 5 × 5 sub-array taken from a total array of 250 × 250 patterns recorded. The 1.5 mrad scale bar applies to all patterns shown. The cut-off at high angle is due to the shadow of the annular dark field detector.

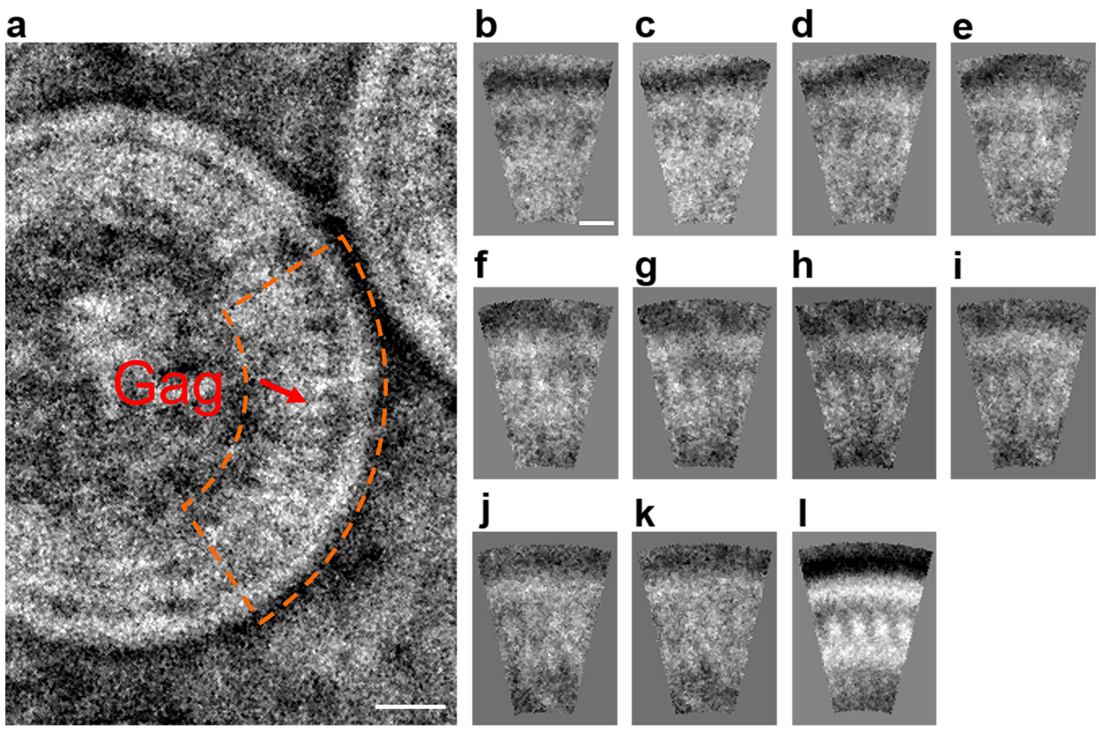

Supplementary Figure 10

Rotational average for Gag protein of HIV-1 VLPs.

(**a**) Sub-area of the reconstructed phase of HIV-1 VLPs shown in **Fig. 3d**. (**b** to **k**) Angular units for averaging selected from the sub-region indicated by the orange arc. (**l**) Rotational average from all the units shown in (**b** to **k**). Scale bars: 20 nm in (**a**); 10 nm in (**b** to **l**).

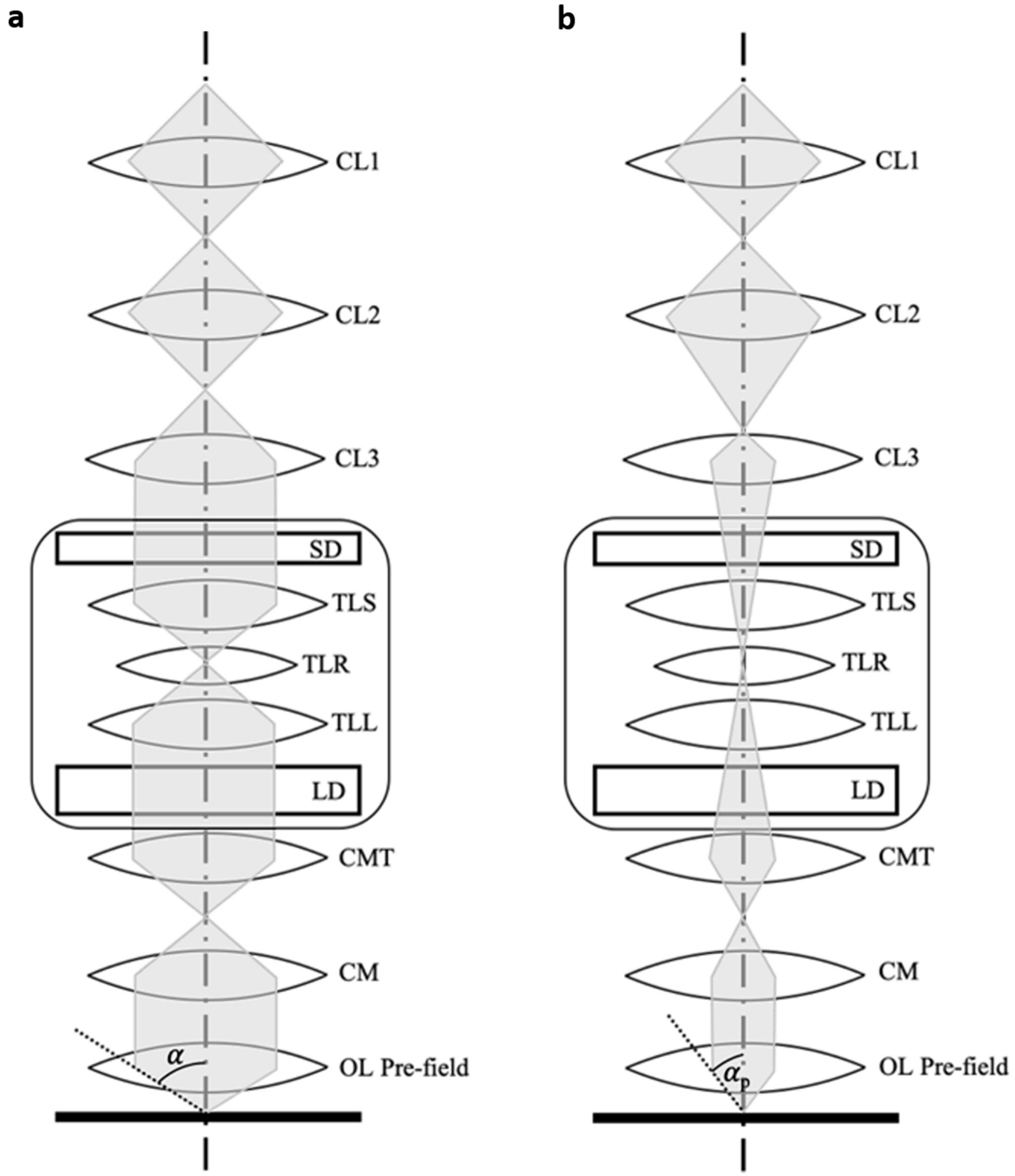

Supplementary Figure 11

Schematic of the illumination optics used in for experimental electron ptychography on the JEOL ARM 300CF compared to normal STEM operation. (**a**) Normal STEM mode. (**b**) Pencil beam mode with the aberration corrector turned off.

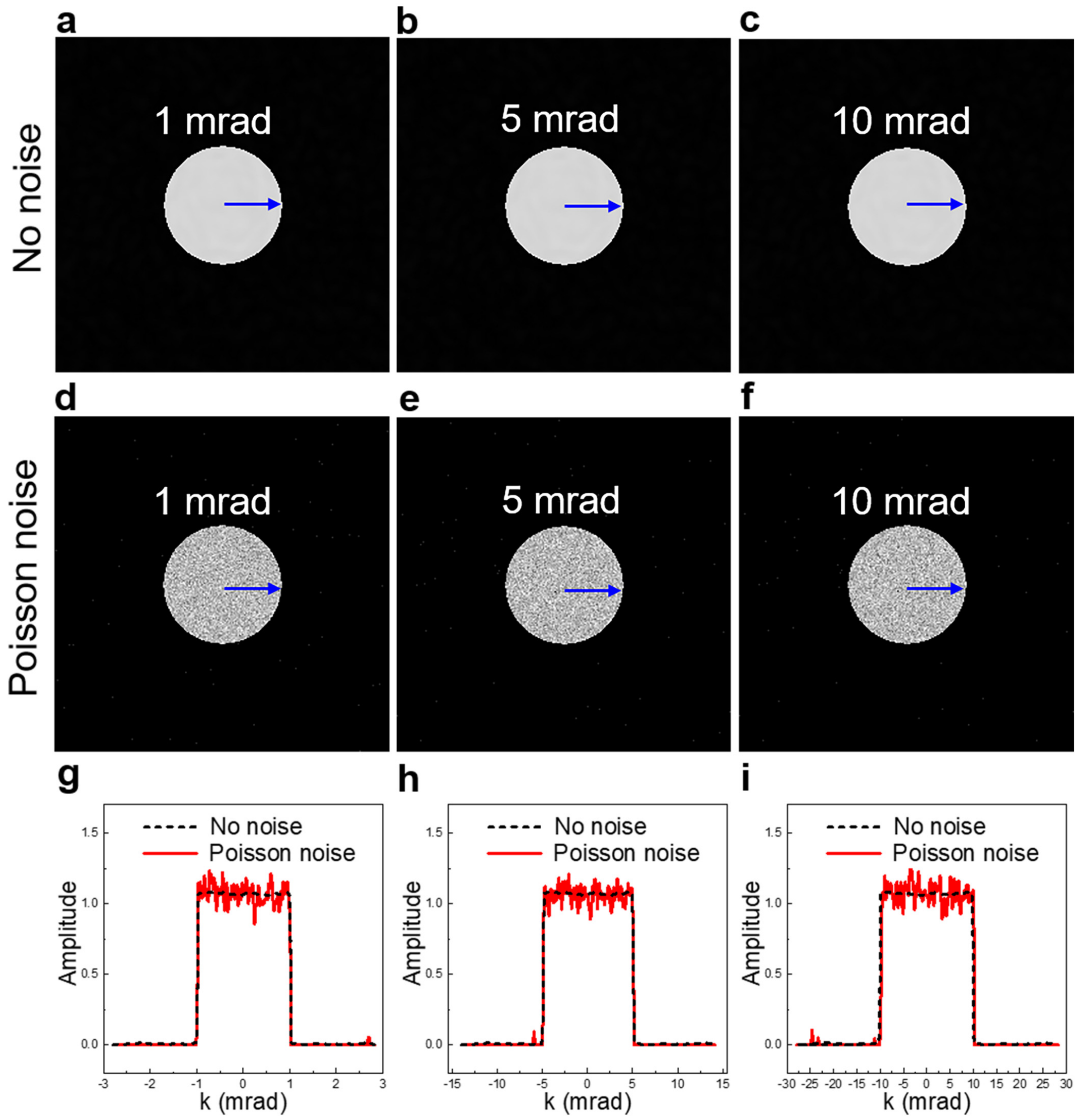

Supplementary Figure 12

Simulated diffraction patterns for different convergence semi-angles.

(**a** to **c**) No noise, (**d** to **f**) With added Poisson noise as described in Supplementary Note 3, (**g** to **i**) Corresponding line profiles across the middle of the diffraction patterns with (red lines) and without noise (black dashed lines).

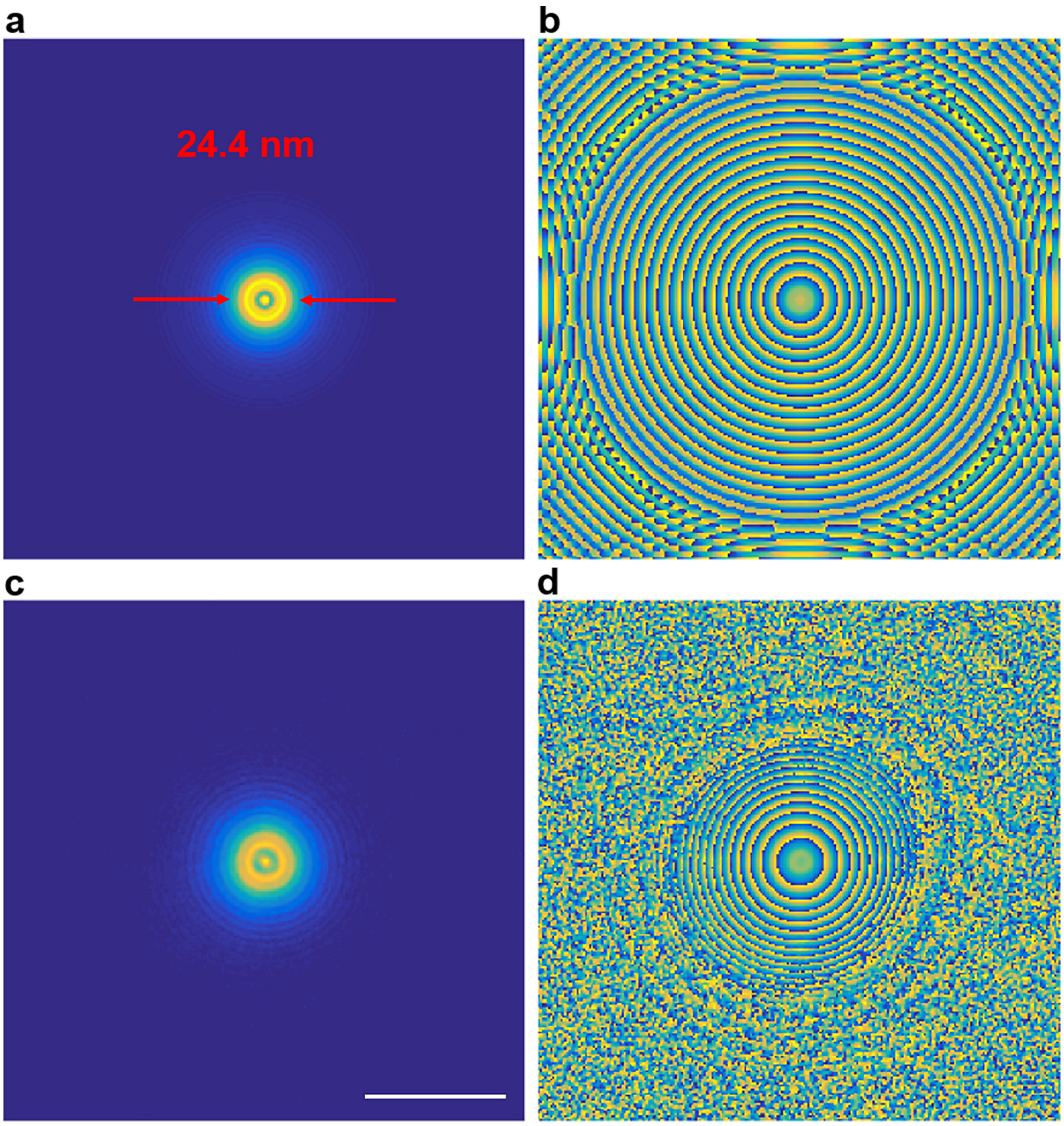

Supplementary Figure 13

Estimated and reconstructed probe functions.

(**a**) Modulus and (**b**) phase of the initial estimated probe function. (**c**) Modulus and (**d**) phase of the probe function after reconstruction. Scale bar: 50 nm.

Supplementary Table 1. Parameters for Cryo-ptychographic data acquisition

	**Setting 1**	**Setting 2**	**Setting 3**
**Accelerating voltage (kV)**	300	300	300
**Convergence semi-angle (mrad)**	1.03	1.03	1.03
**Maximum detection semi-angle (mrad)**	2.18	2.18	2.18
**Sampling in real space (nm)**	0.465	0.465	0.465
**Defocus (µm)**	-13.0	-13.5	-13.5
**Probe size (nm)**	26.9	27.9	27.9
**Diffraction pattern dimension (pixels)**	256×256	256×256	256×256
**Step size (nm)**	3.13	3.13	3.13
**Raster scan dimension**	127×127	127×127	127×127
**Beam current (pA)**	4	2	1
**Dwell time per diffraction pattern (ms)**	1	1	1
**Overlap ratio**	0.85	0.86	0.86
**Dose (e/Å^2)**	22.8	11.3	5.7
**Redundancy σ_{pty}**	185	217	217

Supplementary Table 2. Parameters used for CTF_{Pty} simulations

	**Setting 1**	**Setting 2**	**Setting 3**
**Accelerating voltage (kV)**	80	80	80
**Convergence semi-angle (mrad)**	1	5	10
**Maximum detection semi-angle (mrad)**	2.89	14.22	28.44
**Sampling in real space (nm)**	0.735	0.147	0.073
**Defocus (µm)**	-12.0	-0.485	-0.125
**Diffraction pattern dimension (pixels)**	256×256	256×256	256×256
**Step size (nm)**	2.2	0.42	0.22
**Raster scan dimensions**	300×300	300×300	300×300
**Overlap ratio**	0.88	0.88	0.88
**Redundancy σ_{pty}**	1798	1798	1798

Supplementary Table 3. Parameters for conventional TEM CTF simulation

	Setting 1	Setting 2	Setting 3
**Accelerating voltage (kV)**	80	80	80
**C_s (µm)**	2.3	2.3	2.3
**C_c (mm)**	2.8	2.8	2.8
**Energy spread (eV)**	0.3	0.3	0.3
**Focal spread (nm)**	5.31	5.31	5.31
**Defocus (µm)**	-0.3	-1.0	-2.8

Supplementary Table 4.

Parameters for ptychographic data acquisition at room temperature

	Setting 1	Setting 2	Setting 3	Setting 4
**Accelerating voltage (kV)**	80	80	80	80
**Convergence semi-angle (mrad)**	1.37	4.68	10	1.02
**Maximum detection semi-angle (mrad)**	2.82	11	21	2.89
**Sampling in real space (nm)**	0.74	0.19	0.10	0.72
**Defocus (μm)**	-10.5	-0.80	-0.39	-12.0
**Probe size (nm)**	28.9	7.48	7.80	24.4
**Diffraction pattern dimension (pixels)**	256×256	256×256	256×256	256×256
**Step size (nm)**	1.72	0.8	0.8	4.43
**Raster scan dimension**	126×126	255×255	255×255	250×250
**Beam current (pA)**	6.7	2	1.5	6.7
**Dwell time per diffraction pattern (ms)**	1.3	1.3	1.3	1.3
**Overlap ratio**	0.92	0.86	0.87	0.77
**Dose (e/Å^2)**	146	234	175	27
**Redundancy σ_{pty}**	462	611	206	191

Supplementary Notes

Supplementary Note 1: Experimental Measurements

Cryo-ptychography of rotavirus DLPs

Vitrified TEM samples were loaded into a Gatan 698 Elsa cryo-transfer holder and transferred to a JEOL ARM 300CF operated at 300 kV for electron ptychography experiments, during which the specimen temperature was maintained at -176 ± 2 °C. The cryo-electron ptychography experiments used a STEM imaging mode with a convergence semi-angle of 1.03 mrad. Ptychographic datasets were acquired on a 256 × 256 pixel Merlin Medipix3 direct electron detector[1] with the probe rastered over regions of interest in a grid of 127 × 127 probe positions with a lateral step size of 3.13 nm. The effective pixel size was 0.017 mrad at the detector plane, for a collection semi-angle of 2.18 mrad. The acquisition time for one diffraction pattern was set to 1 ms. Beam currents of 4 pA, 2 pA and 1 pA were used for both rotavirus DLPs and immature HIV-1 VLPs. For both the rotavirus DLPs and immature HIV-1 VLPs these probe currents corresponded to electron doses of 22.8 e/Å^2, 11.3 e/Å^2, 5.7 e/Å^2 for probe diameters of 26.9 nm (defocus= -13.0 μm), 27.9 nm (defocus= -13.5 μm) and 27.9 nm (defocus= -13.5 μm), respectively. The method used to calculate the dose was that given in Ref[2]. Full parameters for the collection of the cryo datasets are given in Supplementary Table 1.

Room temperature ptychographyof an Adenovirus-infected cell embedded in resin

Datasets from an Adenovirus infected cell (Fig. 4a, Supplementary Fig. 8a) and Adenovirus particles (Supplementary Figs. 3a-c) were acquired using a JEOL ARM 300CF operated at 80 kV with a 256 × 256 pixel Merlin Medipix3 direct electron detector[1]. The dataset for the μm size reconstruction of an Adenovirus infected cell was acquired with a convergence semi-angle of 1.02 mrad, a step size of 4.43 nm, a probe size of 24.4 nm, raster scan dimensions of 250 × 250 pixels with an acquisition time of 1.3 ms for one diffraction pattern at a beam current of 6.7 pA, corresponding to a dose of 27 e/Å^2 (Supplementary Table 4, Setting 4). Probes with convergence semi-angles

of 1.37, 4.68 and 10 mrad corresponding to diameters of 28.9 nm, 7.48 nm and 7.80 nm were rastered with step sizes of 1.72 nm, 0.8 nm and 0.8 nm, respectively. The raster dimensions for these three settings were 126 × 126, 255 × 255 and 255 × 255 pixels, respectively. The acquisition time for one diffraction pattern was set to 1.3 ms. Beam currents of 6.7 pA, 2 pA and 1.5 pA were used corresponding to doses of 146 e/Å^2, 234 e/Å^2, 175 e/Å^2. Full details of the parameters for these three acquisitions are given in Supplementary Table 4 (Settings 1-3).

Electron ptychography illumination optics

A schematic of the illumination optics for the electron ptychography experiments on the JEOL ARM300CF compared to normal STEM operation is shown in Supplementary Fig. 11. A pencil beam with small convergence semi-angle was formed using the pre-sample optics of the microscope (Supplementary Fig. 11b). To fine-tune the convergence semi-angle of the probe at the specimen plane, the CL2 cross-over position was adjusted by strengthening the CL2 lens whilst weakening the CL3 lens. All round transfer lenses in the probe corrector were turned off. In contrast conventional STEM uses a large convergence semi-angle (α) compared to that used in this work (α_p for a pencil beam). CL1, CL2 and CL3 represent the condenser lens system, while SD (small dodecapole), TLS, TLR, TLL (transfer lenses) and LD (long dodecapole) are the optical elements in the probe-corrector which are unused and not necessary for this ptychographic optical configuration. The pre-specimen condenser mini-lens system (CMT and CM) and the objective lens pre-field are also shown.

Supplementary Note 2: Sample preparation

Cryo-EM preparation of rotavirus double layered particles (DLPs)

Rotavirus DLPs (strain SA11) were prepared as previously described[3], except that the particles were banded once on a cesium chloride gradient and then diluted in 50 mM Tris HCl, 5 mM EGTA and centrifuged at 100,000 g for 2 h, before being resuspended in 20 mM Tris HCl, 1 mM EGTA. A suspension of rotavirus DLPs was diluted with colloidal AuNPs ~ 6 nm in diameter in 10 mM Tris HCl pH 8.0. Quantifoil holey carbon EM grids were plasma-cleaned for 30 s and four microliters of the DLPs/AuNPs suspension with a concentration of 8 mg/ml was placed onto each EM grid. These were then blotted for 5 s before being plunged into a liquid ethane/propane mixture using a Gatan CP3 semi-manual plunger at a humidity of 80 %. The grids were then transferred and stored under liquid nitrogen.

Cryo-EM preparation of immature HIV-1 VLPs

Immature HIV-1 virus-like-particles (VLPs) were isolated from particles produced by transfection of HEK293T cells with a codon-optimized HIV-1 Gag expression plasmid (‘Gag-opt’). PEI was used for transfecting Gag-opt into HEK293T cells which were grown in Dulbecco’s modified Eagle’s medium supplemented with 10 % fetal calf serum (FCS; Biochrom) in the presence of Pen Strep (0.1 mg/ml). At 48 h post transfection, tissue culture supernatant was harvested and filtered through 0.45 µm nitrocellulose filters. The viral concentration was enriched by ultracentrifugation (120,000 g for 3 h at 4 °C using a Beckman rotor SW32Ti) through a cushion of 20 % (w/w) sucrose in phosphate buffered saline (PBS) buffer. A pellet of HIV-1 VLPs was resuspended with PBS and centrifuged at 125,000 g for 30 minutes at 4 °C with a Beckman TLA55 rotor to remove sucrose. HIV-1 VLPs were resuspended with PBS. The concentration of VLPs was measured by P24 ELISA and Coomassie blue staining by comparing with known BSA as reference.

Quantifoil holey carbon EM grids were glow-discharged for 45 s. The HIV-1 VLP solution was diluted with ~ 10 nm colloid AuNPs in PBS buffer. Cryo-EM grids were

prepared using an FEI Vitrobot with the temperature set to 22 °C and humidity set to 100 %. A 3.5 µl drop of sample solution was added onto the glow-discharged EM grid and then blotted for 5 s before being plunge frozen in liquid ethane. The grids were then transferred and stored under liquid nitrogen.

Preparation of TEM grids of Adenovirus-infected cells embedded in resin

Human Adenovirus type 5 (Ad5) was inoculated on human embryo kidney cells 293 (HEK293) at 0.01MOI (Multiplicity of infection) for 3 days in an incubator at 37 ℃ and 5 % CO_2. Cells were centrifuged at 800 g for 10 min to form a pellet after the cell monolayer was removed from the petri dish. Cell pellets were fixed in 2.5 % glutaraldehyde + 2 % paraformaldehyde buffered by 0.1 M cacodylate (pH 7.4) for 2 h, and rinsed in 0.1 M cacodylate (pH 7.4) three times. The cell pellet was then fixed using 1 % osmium tetroxide (OsO_4) buffered by 0.1 M cacodylate (pH 7.4) for 1h, then rinsed in H_2O three times. The cell pellet was then dehydrated by gradual ethanol as follows:

(1) 50 % ethanol 10 min

(2) 70 % ethanol 10 min

(3) 90 % ethanol 10 min

(4) 100 % ethanol 10 min

(5) 100 % ethanol 10 min

(6) 100 % acetone 10 min

Finally, the cell pellet was infiltrated with PON812 resin as follows:

(1) acetone : resin = 1 : 1 2 h

(2) acetone : resin = 1 : 3 2 h

(3) resin 2 h

After infiltration, the cell pellet was embedded in a module 3 # capsule and the embedded cell pellet was polymerized at 60 ℃ for 24 h to form resin blocks. The resin block was blade trimmed to reveal the cell surface and ultra-thin sections with a nominal thickness of 20 nm were cut using an ultramicrotome (Leica UC7). Sections

were collected on 300 mesh Quantifoil holey carbon EM grids. Finally, the TEM grids were air-dried.

Supplementary Note 3: CTF calculations with and without noise

Ptychographic reconstruction

The easiest way to measure the CTF is to compare power spectra of a measured phase image of an object against that of the known object. Specifically, CTF_{Pty}, the phase CTF for ptychographic reconstruction can be evaluated as:

$$\mathrm{CTF}_{Pty} = \left| \mathrm{FT}(\mathrm{phase}(obj_{recon})) \middle/ \mathrm{FT}(phase(obj_{true})) \right| \tag{1}$$

where FT represents a Fourier transform.

A two-dimensional amorphous phase object function with random phase as shown in Supplementary Fig. 2a with constant amplitude of 1 was generated to represent an original object function (obj_{true}) which was subsequently used to simulate the diffraction patterns for ptychographic reconstruction. The power spectra of the original object function shown in Supplementary Fig. 2b has an approximately uniform intensity distribution across the entire frequency domain. Based on the simulated diffraction patterns from the original object function, the simulated reconstructed ptychographic phase (obj_{recon}, as shown in Supplementary Fig. 2c as an example) were reconstructed using the ePIE algorithm. The power spectra of the reconstructed phase of obj_{recon} (Supplementary Fig. 2f) show the intensity distribution of the resultant phase information across the frequency domain transferred by the ePIE reconstruction. To evaluate how strongly the ePIE ptychographic algorithm transfers the phase information of the original object (obj_{true}) to the reconstructed one (obj_{recon}) as a function of spatial frequency, the ratio between the amplitudes of the power spectra of obj_{recon} and obj_{true} phases can be calculated using Eq. 1. Plots of CTF_{pty} as shown in Supplementary Fig. 2o can be subsequently calculated by radially averaging this ratio map.

The parameters used for the CTF_{Pty} simulations shown in Fig. 2 are given in Supplementary Table 2. For example, Settings (1-3), used convergence semi-angles of 1 mrad, 5 mrad and 10 mrad with defoci of -12.0 µm, -0.485 µm and -0.125 µm respectively for the calculations of CTF_{Pty} at 80kV. To maintain an overlap ratio of 88 %

and a redundancy of 1797 for all convergence semi-angles, a 300 × 300 array of diffraction patterns each recorded using 256 × 256 pixels were simulated with step sizes of 2.2 nm, 0.42 nm and 0.22 nm, respectively. Typical calculated diffraction patterns without noise for convergence semi-angles of 1 mrad, 5 mrad and 10 mrad are shown in Supplementary Figs. 12a-c. Using these diffraction patterns, the phase of the ptychographic object functions (obj_{recon}) for these convergence semi-angles (shown in Supplementary Figs. 2c-e) were reconstructed using ePIE, with their corresponding power spectra shown in Supplementary Figs. 2f-h. Subsequently, CTF_{Pty} without noise (Supplementary Fig. 2o) was calculated using Eq. 1.

For the experiments reported, diffraction patterns were recorded in an electron counting detector at low dose. Hence, the main experimental detector noise can be considered as Poisson distributed shot noise, modelled as follows.

Assuming independent measurements $I_{n,i}$ at the i-th pixel on the detector in the n-th illumination of electron probe, the probability that the entire ptychographic data set I is collected is[4]:

$$f_p(I) = \prod_n \prod_i e^{-\lambda_{n,i}} * \frac{[\lambda_{n,i}]^{I_{n,i}}}{I_{n,i}!} \quad (2)$$

where $\lambda_{n,i}$ is the expected number of events for the i-th pixel in the n-th illumination. Adding Poisson distributed shot noise, the detected intensity $I_{n,i}$ at i-th pixel in the n-th illumination is given by $Poisson(\lambda_{n,i})$, where $Poisson(\lambda_{n,i})$ denotes the Poisson noise yields and returns a random number from a Poisson distribution with expectation $\lambda_{n,i}$, as in Eq. 2.

For simplicity, Poisson distributed shot noise corresponding to a total incident electron count (58560 e^-) in an experimental diffraction pattern acquired with a convergence semi-angle of 1 mrad at 80 kV, was subsequently added to each simulated diffraction patterns using code written in Matlab[4,5]. Typical calculated diffraction patterns with added Poisson noise for convergence semi-angles of 1 mrad, 5 mrad and 10 mrad are shown in Supplementary Figs. 12d-f. Line profiles extracted across the middle of the diffraction patterns are shown in Supplementary Figs. 12g-i, indicating that the signal-

to-noise (S/N) ratio included in the calculations for all three convergence semi-angles is the same. The phase of the reconstructed ptychographic object functions (obj$_{recon}$) for these three convergence semi-angles were subsequently recovered as shown in Supplementary Figs. 2i-k, respectively with their corresponding power spectra shown in Supplementary Figs. 2l-n. The CTF$_{Pty}$ with noise (Fig. 2a and Supplementary Fig. 2o) was similarly calculated using from Eq. 1.

Without noise in the ptychographic dataset, the phase CTFs can extend to the Nyquist frequency for each convergence semi-angle considered in the calculations, indicating that ptychographic reconstruction uses signal up to the maximum collection angle of the detector, even when the signal is low. However, when noise was introduced the S/N in the dark field region is too low to allow any signal beyond the bright field disc to be reconstructed, limiting the ultimate resolution. As a consequence, the high frequency bound of the noise limited CTF$_{Pty}$ lies at the twice the convergence semi-angle as shown in Fig. 2a and Supplementary Fig. 2o.

Therefore, for the experiments as reported here, the level of the S/N ratio at the detector plane within the collection angle used to record the data can impose a lower resolution limit and specifically in the weak phase approximation, the resolution for iterative ptychography is limited to twice the convergence semi-angle of the probe used.

Conventional TEM

For C_s=2.3 μm, C_c=2.8 mm at an accelerating voltage of 80 kV, CTFs[6] were calculated including an envelope function (ENV) modelling the partial spatial and temporal coherence at a spatial frequency (u) defined as[7-9]:

$$\mathrm{CTF}(u) = \sin(\pi \Delta f \lambda u^2 + \frac{1}{2}\pi C_s \lambda^3 u^4) \quad (3)$$

$$\mathrm{ENV}(u) = \exp[-\{\pi\beta(C_s\lambda^2 u^3 + u)\}^2]\exp\{-(\pi\lambda\Delta)^2 u^4/2\} \quad (4)$$

where, $\Delta = C_c\sqrt{(2\frac{\delta I}{I})^2 + (\frac{\delta E}{E})^2 + (\frac{\delta V}{V})^2}$

The CTF was multiplied with these envelope functions for a beam convergence, β=0.1 mrad and focal spread of 5.31 nm. The energy spread of the electron beam, δV=0.3 eV

for the cold field emission gun (CFEG) used. The current instability of the objective lens is $\delta I/I$ =0.0000005, and the instability of the accelerating voltage is $\delta E/E$ =0.0000005. CTFs were calculated at defoci (Δf) -0.3 μm, -1.0 μm, -2.8 μm, corresponding to the values used for experimental data acquisition of the Adenovirus particle images shown in Figs. 2d-f.

Supplementary Note 4: Fourier ring correlation

To evaluate the resolution (the high spatial frequency bound) of a ptychographic reconstruction, Fourier ring correlation (FRC)[10,11] was used, which measures the degree of correlation between two images at different spatial frequencies. The full dataset (126 × 126 diffraction patterns) recorded from an Adenovirus particle shown in Supplementary Fig. 3a was split into two independent datasets (63 × 63 diffraction patterns) as illustrated in Supplementary Fig. 3f, for ptychographic reconstruction. The FRC was then calculated from the complex function of these two reconstructions as a function of spatial frequency. To quantify the resolution from the FRC, a threshold criterion of ½-bit was chosen[11]. However, since the independent reconstructions use half the number of electrons and a reduced probe overlap[12], the independent, separate reconstructions are of lower quality than that from the full dataset. Hence, this method provides a conservative estimate of the resolution in the ptychographic reconstruction.

Supplementary Note 5: Conventional phase contrast TEM imaging

Room Temperature TEM of Adenovirus particles embedded in resin

Conventional phase contrast TEM images of Adenovirus particles (Figs. 2d-f and Supplementary Figs. 5a-e were acquired using a JEOL ARM 300CF operated at 80 kV. The Adenovirus particle was imaged across a range of defoci from -0.32 µm to -25 µm, with a fixed spherical aberration coefficient of 2.24 µm. A pixel size of 0.74 nm and a dose of 180 e/Å^2 were used to record the data, similar to those used in the ptychographic reconstruction shown in Fig. 2c (0.74 nm and 146 e/Å^2).

Cryo TEM of DLPs

Movies of rotavirus DLPs in vitrified ice were acquired on a Titan Krios using a Falcon III detector in linear mode with a total exposure of 35 e/Å^2 distributed over 29 motion-corrected fractions.

Supplementary Note 6: Ptychographic reconstruction configurations

Evaluation of the dataset redundancy

For ptychographic reconstruction, the quantity σ_{pty}[13] can be used to estimate the degree of redundancy of a ptychographic dataset as:

$$\sigma_{pty} = \frac{J(\text{total pixels used in diffraction patterns})}{2(\text{pixels in specimen \& probe reconstructions})} \tag{5}$$

where J represents the total number of pixels in the diffraction patterns used for reconstruction. Using Setting (1) in Supplementary Table 1 of Fig. 1b as an example, the reconstructed object consisted of 1539 × 1539 pixels. In this case the diffraction pattern used only the central 236 × 236 pixels. The number of recorded diffraction patterns is 127 × 127 and therefore, the total number of pixels in the diffraction pattern used for reconstruction (J) is $127^2 \times 236^2$, while the number of unknown pixels in the specimen and probe is $2 \times (1539 \times 1539 + 256^2)$. Hence, σ_{pty} is 185, implying that the ptychographic reconstruction is suitably conditioned for this dataset[13].

ePIE ptychographic reconstruction

Ptychographic reconstruction was carried out using the extended ptychographic iterative engine (ePIE)[14,15]. For the cryo datasets of rotavirus DLPs at doses 22.8 e/Å^2 (Fig. 1b and Fig. 3a), 11.3 e/Å^2 (Fig. 3b) and 5.7 e/Å^2 (Fig. 3c), the overlap ratio was 85 %, 86 % and 86 %, respectively, giving a degree of redundancy[13] for the three ptychographic datasets estimated as 185, 217 and 217, with corresponding central pixel arrays of 236 × 236, 256 × 256 and 256 × 256 in the diffraction patterns used for reconstruction. For the cryo datasets of immature HIV-1 VLPs at doses 22.8 e/Å^2 (Fig. 3d), 11.4 e/Å^2 (Fig. 3e) and 5.7 e/Å^2 (Fig. 3f), the overlap ratio was 85 %, 85 % and 86 %, respectively, giving a degree of redundancy[13] for these three ptychographic datasets estimated as 160, 191 and 217, with the corresponding central pixel arrays of 220 × 220, 240 × 240 and 256 × 256 in the diffraction patterns used for reconstruction. For the datasets of the Adenovirus with different convergence semi angles of 1.37 mrad (Supplementary Fig. 3a), 4.68 mrad (Supplementary Fig. 3b) and 10 mrad

(Supplementary Fig. 3c), the overlap ratio was 92 %, 86 % and 87 %, respectively, with central pixel arrays for each diffraction pattern used in the reconstruction of 192 × 192, 256 × 256, 256 × 256, giving corresponding redundancies of 462, 611 and 206, respectively. For the micrometer dataset of Adenovirus infected cell shown in (Fig. 4a), the overlap ratio is 77 % and the redundancy is 191 with the central 194 × 194 pixels in the diffraction pattern used for reconstruction. All probe and object functions shown were obtained after 300 iterations of the ePIE algorithm. The estimated and reconstructed probe functions used for Setting (4) in Supplementary Table 4 are given in Supplementary Fig. 13.

Supplementary Note 7: Rotational average for Gag protein of HIV-1 VLP.

The sub-region, indicated by the orange line in Fig. 3d and Supplementary Fig. 10a, is enclosed by a section over 87.75º with 48.36 nm distance between the inside and outside arcs. The first unit over 27º as shown in Supplementary Fig. 10b, which contains 4 Gag protein particles, is selected from the top of the arc sub-region. Another 9 units, shown in Supplementay Figs. 10c-k, are then selected at intervals of 6.75º successively. Finally, the rotational average shown in Supplementary Fig. 10l of four Gag protein particles from the sub-region is generated by aligning and summing the 10 units.

Supplementary References:

1. Mir, J. A. *et al.* Characterisation of the Medipix3 detector for 60 and 80keV electrons. *Ultramicroscopy* **182**, 44-53 (2017).
2. Song, J. *et al.* Atomic resolution defocused electron ptychography at low dose with a fast, direct electron detector. *Sci. Rep.* **9**, 3919 (2019).
3. Street, J. E., Croxson, M. C., Chadderton, W. F. & Bellamy, A. R. Sequence diversity of human rotavirus strains investigated by northern blot hybridization analysis. *J. Virol.* **43**, 369 (1982).
4. Godard, P., Allain, M., Chamard & V. & Rodenburg, J. Noise models for low counting rate coherent diffraction imaging. *Opt. Express* **20**, 25914-25934 (2012).
5. Huang., X. *et al.* Signal-to-noise and radiation exposure considerations in conventional and diffraction x-ray microscopy. *Opt. Express* **17**, 13541 (2009).
6. Scherzer, O. Über einige Fehler von Elektronenlinsen. *Zeitschrift für Physik* **101**, 593-603 (1936).
7. Hanszen, K. & Trepte, L. Der einfluss von strom-und spannungsschwankungen, sowie der energiebreite der strahlelektronen auf kontrastübertragung und auflösung des elektronenmikroskops. *Optik* **32**, 519-538 (1971).
8. Frank, J. The envelope of electron microscopic transfer functions for partially coherent illumination. *Optik* **38**, 519-536 (1973).
9. Wade, R. H. & Frank, J. Electron-microscope transfer-functions for partially coherent axial illumination and chromatic defocus spread. *Optik* **49**, 81-92 (1977).
10. Saxton, W. O. & Baumeister, W. The correlation averaging of a regularly arranged bacterial cell envelope protein. *J. Microsc.* **127**, 127-138 (1982).
11. van Heel, M. & Schatz, M. Fourier shell correlation threshold criteria. *J. Struct. Biol.* **151**, 250-262 (2005).
12. Bunk, O. *et al.* Influence of the overlap parameter on the convergence of the ptychographical iterative engine. *Ultramicroscopy* **108**, 481-487 (2008).
13. Maiden, A. M., Humphry, M. J., Zhang, F. & Rodenburg, J. M. Superresolution imaging via ptychography. *J. Opt. Soc. Am. A* **28**, 604-612 (2011).
14. Maiden, A. M. & Rodenburg, J. M. An improved ptychographical phase retrieval algorithm for diffractive imaging. *Ultramicroscopy* **109**, 1256-1262 (2009).
15. Wang, P., Zhang, F., Gao, S., Zhang, M. & Kirkland, A. I. Electron ptychographic diffractive imaging of Boron atoms in LaB_6 crystals. *Sci. Rep.* **7**, 2857 (2017).

16. Freed, E. O., HIV-1 assembly, release and maturation. *Nat. Rev. Microbiol.* **13**, 484 (2015).
